# Two redox-responsive LysR-type transcription factors control the oxidative stress response of Agrobacterium tumefaciens

**DOI:** 10.1093/nar/gkaf267

**Published:** 2025-04-07

**Authors:** Janka J Schmidt, Vivian B Brandenburg, Hannah Elders, Saba Shahzad, Sina Schäkermann, Ronja Fiedler, Lisa R Knoke, Yvonne Pfänder, Pascal Dietze, Hannah Bille, Bela Gärtner, Lennart J Albin, Lars I Leichert, Julia E Bandow, Eckhard Hofmann, Franz Narberhaus

**Affiliations:** Microbial Biology, Ruhr University Bochum, 44801 Bochum, Germany; Bioinformatics Group, Ruhr University Bochum, 44801 Bochum, Germany; Protein Crystallography, Ruhr University Bochum, 44801 Bochum, Germany; Ernst Ruska-Centre for Microscopy and Spectroscopy with Electrons (ER-C-3): Structural Biology, Institute of Biological Information Processing (IBI-6): Structural Cell Biology, Forschungszentrum Jülich, 52428 Jülich, Germany; Applied Microbiology, Ruhr University Bochum, 44801 Bochum, Germany; Center for System-based Antibiotic Research, Ruhr University Bochum, 44801 Bochum, Germany; Microbial Biology, Ruhr University Bochum, 44801 Bochum, Germany; Microbial Biochemistry, Ruhr University Bochum, 44801 Bochum, Germany; Microbial Biology, Ruhr University Bochum, 44801 Bochum, Germany; Applied Microbiology, Ruhr University Bochum, 44801 Bochum, Germany; Microbial Biology, Ruhr University Bochum, 44801 Bochum, Germany; Microbial Biology, Ruhr University Bochum, 44801 Bochum, Germany; Microbial Biology, Ruhr University Bochum, 44801 Bochum, Germany; Microbial Biochemistry, Ruhr University Bochum, 44801 Bochum, Germany; Applied Microbiology, Ruhr University Bochum, 44801 Bochum, Germany; Center for System-based Antibiotic Research, Ruhr University Bochum, 44801 Bochum, Germany; Protein Crystallography, Ruhr University Bochum, 44801 Bochum, Germany; Microbial Biology, Ruhr University Bochum, 44801 Bochum, Germany

## Abstract

Pathogenic bacteria often encounter fluctuating reactive oxygen species (ROS) levels, particularly during host infection, necessitating robust redox-sensing mechanisms for survival. The LysR-type transcriptional regulator (LTTR) OxyR is a widely conserved bacterial thiol-based redox sensor. However, members of the Rhizobiales also encode LsrB, a second LTTR with potential redox-sensing function. This study explores the roles of OxyR and LsrB in the plant-pathogen *Agrobacterium tumefaciens*. Through single and combined deletions, we observed increased H_2_O_2_ sensitivity, underscoring their function in oxidative defense. Genome-wide transcriptome profiling under H_2_O_2_ exposure revealed that OxyR and LsrB co-regulate key antioxidant genes, including *katG*, encoding a bifunctional catalase/peroxidase. *Agrobacterium tumefaciens* LsrB possesses four cysteine residues potentially involved in redox sensing. To elucidate the structural basis for redox-sensing, we applied single-particle cryo-EM (cryogenic electron microscopy) to experimentally confirm an AlphaFold model of LsrB, identifying two proximal cysteine pairs. *In vitro* thiol-trapping coupled with mass spectrometry confirmed reversible thiol modifications of all four residues, suggesting a functional role in redox regulation. Collectively, these findings reveal that *A. tumefaciens* employs two cysteine-based redox sensing transcription factors, OxyR and LsrB, to withstand oxidative stress encountered in host and soil environments.

## Graphical Abstract

**Graphical Abstract:**
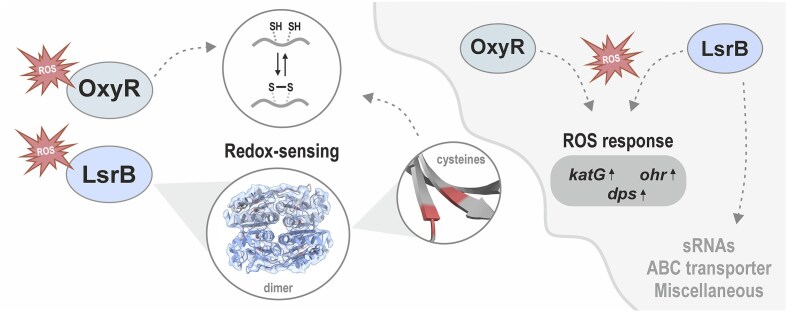

## Introduction

Facultative pathogens, like the phytopathogen *Agrobacterium tumefaciens* alternate between free-living and host environments. Efficient adaptation to readily changing environmental perturbations, such as fluctuations in reactive oxygen species (ROS) requires rapid sensing and coordinated responding. Endogenous ROS are continuously and inevitably generated as a byproduct of aerobic respiration [1, 2]. During host pathogen interactions, invading pathogens are additionally exposed to exogenous ROS, released by the eukaryotic hosts in response to infection [1].

The adaptive response to ROS is mediated by complex, regulatory networks consisting of redox-sensing regulators, detoxification-, and repair systems [1]. Primarily, cellular detoxification systems include superoxide dismutases (SODs), which convert superoxide anion radicals (·O_2_^−^) to molecular oxygen and hydrogen peroxide (H_2_O_2_). H_2_O_2_ is then readily detoxified to molecular oxygen and water by catalases or peroxidases [3]. As the highly reactive hydroxy radicals (OH·) are generated in the Fenton reaction from H_2_O_2_ in the presence of free iron (Fe^2+^), limiting the levels of free intracellular iron plays a key role in maintaining the cellular redox homeostasis [4, 5]. In addition, the small molecular weight thiol-containing tripeptide glutathione is an important redox buffer in many organisms with a cytosolic concentration of up to 5–10 mM [6] and a ratio of reduced (GSH) to oxidized glutathione disulfide (GSSG) of 65′000:1 in *Escherichia coli* [7]. GSH either directly scavenges oxidants and thereby protects cytosolic thiols from oxidation or serves as electron donor for glutaredoxins (Grx) that reduce nonconsecutive disulfide bonds in cytosolic proteins [8]. Once oxidized, GSSG can be reduced by the glutathione reductase using NADPH as electron source [9]. The redox homeostasis is disturbed when stress response systems are unable to cope with the amount of endogenously produced or host-released ROS, which creates oxidative stress [1, 10–12]. In turn, ROS severely damage lipids, nucleic acids, and proteins, which impacts their structure and function, eventually leading to toxic effects and ultimately cell death.

In the cytosol, protein thiols are maintained in a reduced form by the thioredoxin or glutaredoxin systems [13]. However, cysteine residues are susceptible to post-translational, oxidation-dependent reversible or irreversible oxidative thiol modifications [14–17]. A reversible thiol modification is e.g. the initial product of the oxidation of a thiol group by H_2_O_2_ to a highly reactive sulfenic acid intermediate (S-OH). Sulfenic acid rapidly reacts with nearby thiol groups, leading to the formation of intra- or intermolecular disulfide bonds [17–19]. Additionally, low-molecular-weight thiols can react with sulfenic acids to form mixed disulfides, also known as S-thiolations [20]. These modifications are reversible e.g. through the thioredoxin or glutaredoxin system [21–23]. However, elevated ROS levels can lead to overoxidation of protein thiols to cysteine to sulfinic or sulfonic acids, both irreversible modifications [17]. Reversible and irreversible thiol modifications likely result in structural and/or functional changes or eventually loss of function.

The unique *in vivo* reversibility of cysteine oxidation products with sulfur in its lower oxidation state forms the molecular basis of thiol-based redox regulation. This mechanism allows redox-responsive regulators, to utilize this thiol-based redox switch to directly sense and respond to changes in the cell’s redox state [17–19]. Through reversible thiol oxidation, these switches can be activated in response to oxidative stress and subsequently inactivated once redox balance is restored. Consequently, thiol-based redox switches orchestrate the genetic response to oxidative stress. Notably, many oxidative stress response regulators are functionally conserved, despite the large genomic diversity of bacteria. A well-characterized example is the H_2_O_2_ regulator OxyR (Atu4641 in *A. tumefaciens*) [24]. OxyR is a member of the LysR-type transcriptional regulator (LTTR) family, present in Proteobacteria, Bacteriodetes, and Actinobacteria. A redox-active cysteine residue (C199 in *E. coli*) located in the C-terminal co-effector binding domain is responsible for OxyR activation [24–33]. In turn, OxyR activates the transcription of various detoxification systems, such as catalases.

Unlike many bacteria, *Agrobacterium* harbors another redox-associated LTTR, called LsrB (LysR-type symbiosis regulator B). LsrB is solely found in Rhizobiales, such as the plant symbiont *Sinorhizobium meliloti* or the intracellular mammalian pathogen *Brucella abortus* (there designated as VtlR = virulence-associated transcriptional LysR-family regulator). LsrB is a major regulator of various cellular responses, including the coordinated regulation of small RNAs, ABC transporter or β-lactam resistance genes [34–37]. Notably, in *S. meliloti* it was shown that LsrB is responsible for the coordinated regulation of the glutathione and catalase genes, as well as positively regulating OxyR [38, 39]. A previous study identified three cysteine residues in the co-effector binding domain of *S. meliloti* LsrB, which were found to be crucial for both target gene regulation and the oligomerization of this transcription factor, suggesting a potential thiol-based activation mechanism [39]. It was proposed that the cysteines sense oxidative stress via the formation of intermolecular disulfide bonds.

In the present study, we investigate the mechanisms driving LsrB’s response to oxidative stress and its interplay with the H_2_O_2-_responsive regulator OxyR in *A. tumefaciens*. Transcriptome profiling via RNA-sequencing (RNA-seq) under H_2_O_2_ stress revealed that both LsrB and OxyR positively control central antioxidant genes, correlating with increased H_2_O_2_ sensitivity of single or double deletion of the regulators. Intriguingly, deletion of *oxyR* specifically increased H_2_O_2_ sensitivity, while *lsrB* deletion led to increased susceptibility to a broader spectrum of ROS, including paraquat and oxidized glutathione. To further support that LsrB acts as thiol-based redox switch, we examined its structure by AlphaFold 3-based modeling and single-particle cryo-EM. This revealed that the four cysteine residues in *Agrobacterium* LsrB form two distinct pairs, strongly suggesting intramolecular disulfide bond formation. This was further supported by a mass spectrometry-based thiol-trapping approach. Our results show that *Agrobacterium* uses two redox-responsive LTTRs to coordinate the oxidative stress response.

## Materials and methods

### Bacterial strains, plasmids, oligonucleotides, and media

A detailed list of all plasmids, oligonucleotides and bacterial strains used in this study is summarized in the supplemental material (Supplementary Tables S1-S3). *Escherichia coli* DH5α and JM83 served as hosts for construction and subsequent storage of plasmids. For recombinant protein production using the pET-expression system, *E. coli* BL21 (DE3) was used. Strains were cultivated in Lysogeny broth (LB) medium, supplied with kanamycin (50 μg/ml), chloramphenicol (50 μg/ml), or ampicillin (100 μg/ml) at 37°C until an optical density (OD_600_) of 0.5–0.8 was reached and induced with 1 mM Isopropyl β-D-1-thiogalactopyranoside (IPTG) and cultivated overnight at 20°C. *Agrobacterium tumefaciens* strains were cultivated in LB medium at 30°C*. Agrobacterium tumefaciens* strains carrying the pTrc200 or pSRK derivate were cultivated in LB medium containing appropriate antibiotics (100 μg/ml streptomycin and 300 μg/ml spectinomycin or 50 μg/ml kanamycin). Strains carrying pTrc200 derivates (with *katG*) were induced with 1 mM IPTG. LB medium was prepared with 0.5% (w/v) yeast extract, 1% (w/v) tryptone, and 1% (w/v) NaCl. Strains carrying the pLK23 were grown in AB minimal medium [37] and protein production was induced using 0.2 mM IPTG and growth at 20°C overnight.

### Construction of a markerless *A. tumefaciens oxyR* (*atu4641*) mutant

The marker less *oxyR*-deletion strain was constructed as previously described [40]. Here, two DNA fragments corresponding to upstream (332 bp) and downstream regions (384 bp) of *oxyR* (*atu4641*) were amplified by polymerase chain reaction (PCR) with appropriate primers (listed in Supplementary Table S3). Fragments were ligated and cloned into the suicide vector pK19*mobsacB*. The resulting plasmid (pJS51) was transferred into *A. tumefaciens* wild-type (WT) or the *ΔlsrB* knock out strain (for double deletion) by biparental mating according to Klipp *et al.* [41]. Positive clones were selected as previously described [42].

### Construction of catalase-boost plasmids

A PCR-derived 6x-His tag was added to the C-terminus of *katG* (*atu4642*) and introduced to the pTrc200 via standard restriction and ligation protocols. The primers used are listed in Supplementary Table S3. The resulting plasmid was transferred into *A. tumefaciens* strains by electroporation and selected by streptomycin and spectinomycin resistance. Successful expression of *katG* was further validated by western blot analysis, using a penta-his tag antibody.

### Construction of roGFP2 expression plasmids

The pTrc200 vector backbone was used to generate roGFP2 expression plasmids. The gentamicin cassette (853 bp) was excised using NcoI and XbaI. *grx1*-*roGFP2* was PCR amplified from pCC-*grx1*-*roGFP2*^His^ [43] and integrated into the pTrc200 vector backbone via the primer-derived restriction start sites Nco and XbaI. Plasmids were verified by sequencing with a sequence specific primer. The primers used are listed in Supplementary Table S3. The resulting plasmid was transferred into *A. tumefaciens* strains by electroporation and selected by streptomycin and spectinomycin resistance. Grx1-roGFP expression was verified by western blot analysis using GFP-specific monoclonal antibody before further use.

### Site-directed mutagenesis of *lsrB* (*atu2186*)

Mutagenesis of *lsrB* was achieved by site-directed mutagenesis using the Q5^®^ Site-Directed Mutagenesis Kit (New England Biolabs) using the *lsrB* complementation plasmid [36]. Primer pairs used are listed in Supplementary Table S3. Successful mutagenesis was verified by sequencing. In total 11 plasmids were constructed carrying single, double or triple cysteine substitutions, with Ser respectively (listed in Supplementary Table S2). Successful mutagenesis was verified by sequencing using the M13 standard primer, provided by MicroSynth Seqlab. For the generation of a quadruple cysteine substitution, a *lsrB* fragment carrying the mutations and restriction sites for BamHI and SacI was synthesized via Twist Bioscience and integrated into the pSRK vector by standard restriction digest and ligation. The generated plasmids were transferred into *A. tumefaciens* by electroporation and selected by kanamycin resistance.

### Determination of the steady state redox potential exerted on Grx1-roGFP2

The steady state redox potential of Grx1-roGFP2 in *A. tumefaciens* WT and LsrB deficient cells by expression from pTrC-*grx1*-*roGFP2* plasmid was determined as described previously for roGFP2 in *E. coli* [44]. Correct localization and expression were confirmed by fluorescence microscopy. To determine the oxidation state of Grx1-roGFP2, cells expressing Grx1-roGFP2 as described above for 16 h at 20°C were harvested by centrifugation, washed twice in phosphate-buffered saline (PBS) buffer (1.5 mM KH_2_PO_4_, NaCl 150 mM, 2.7 mM Na_2_HPO_4_-7xH_2_O, pH 7.4) and adjusted to an OD_600_ of 1.0 in PBS. Then, fluorescence intensities were recorded as described previously [45] with emission fixed at 510 nm (±5 nm) every 30 s for at least 3 min at 20°C in an FP-8500 spectrofluorometer (Jasco, Tokyo, Japan) with continuous stirring. Excitation was scanned from 350 to 500 nm with 5 nm bandwidth and medium sensitivity. Cells treated with 5 mM aldrithiol-2 (AT-2) or 20 mM dithiotreitol (DTT) served as controls for full oxidation and reduction of the probe. The fluorescence excitation ratios (405/488 nm) were calculated to determine the probe’s oxidations state (OxD) using equation [X] and the steady state redox potential using the Nernst equation [y] as described previously [45, 46].

Equation [x]


\begin{eqnarray*}
OxD = \frac{{R - {R_{red}}}}{{\left( {\frac{{I_{ox}^{488}}}{{I_{red}^{488}}}} \right)*\left( {{R_{ox}} - R} \right) + \left( {R - {R_{red}}} \right)}}
\end{eqnarray*}

*R_ox_* was 405/488 nm ratio of AT-2- (oxidized) and *R_red_* of DTT- (reduced). *I_488_ox and I_488_red* are the fluorescence intensities of Grx1-roGFP2 at 488 nm under oxidizing or reducing conditions. *R* is the measured 405/488 nm ratio of the probe. All values were the means from three time points before (UT) or after addition of the oxidant (AT-2) or reductant (DTT).

Nernst equation [y]:


\begin{eqnarray*}
{E_{Grx1 - roGFP2}} &=& E_{Grx1 - roGFP2}^0 \nonumber\\ &-& \frac{{R*T}}{{n*F}}*\ln \left( {\frac{{1 - OxD}}{{OxD}}} \right)
\end{eqnarray*}

with *E^0^_Grx1-oGFP2_* = −280 mV [https://doi.org/10.1038/nmeth.1212], *R* = 8.314 J.K^−1^mol^−1^ (gas constant), *T* (293.15 K 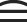 20°C), *n* = number of transferred electrons (2), and *F* = Faraday’s constant (96 485 C mol^−1^).

### Isolation of total RNA

Total RNA was isolated from *A. tumefaciens* cultures (10 ml) grown in LB-medium to mid-exponential phase (OD_600_= 0.8). Samples were collected before and after treatment with 5 mM H_2_O_2_ for 15 min and RNA was extracted via the hot acid phenol method, as previously described [42, 47]. Residual chromosomal DNA was removed via the TURBO DNA free kit (Thermo Fischer Scientific) according to the manufacture’s instruction. DNA removal was validated by PCR.

### Quantitative Reverse Transcription Polymerase Chain Reaction (qRT-PCR)

1 μg of DNA-free RNA was used for reverse transcription via the LunaScript RT (New England Biolabs) according to the protocol provided by the manufacturer. The CFX Connect^TM^ Real-Time system (Bio-Rad Inc.) was used to perform qRT-PCR, as described previously [36, 37]. Differential expression was calculated using the ΔC_T_ method. The gene *gyrB* (atu0012) served as reference gene for transcript level normalization.

### RNA-sequencing

Library preparation and sequencing on the Illumina NovaSeq 6000 platform was performed by Novogene Co., Ltd. Data analysis and statistical evaluation was performed according to our previously described protocol [36]. Here, genes with adjusted *P* < .01 and >2-fold up- or downregulation relative to WT were considered as differentially expressed. The nomenclature used for small RNAs was adapted from [48]. Genomic localization is given as C1 = circular chromosome; C2 = linear chromosome; pAt = At-plasmid or pTi = Ti-plasmid. Transcription start sites are indicated as F = forward strand or R = reverse strand. The RNA-seq data are deposited at the at Gene Expression Omnibus under the accession number GSE283062.

### Gene ontology enrichment analysis

Gene ontology (GO) terms (NCBI taxon-ID 176299, protein enrichment terms v12.0) were downloaded from STRING [49]. The GO enrichment for all genes with a log_2_ fold change > 1 was conducted with topGO with the ‘weight01’ algorithm [50, 51] and Fisher’s exact test. Custom scripts for preprocessing as well as visualization can be found at the GitHub repository https://github.com/VivianBrandenburg/rnanalysis.

### Protein purification

His-tagged ArgP and His-tagged or Strep-tagged LsrB were overexpressed using *E. coli* BL21 (DE3). His-tagged proteins were purified as previously described [37]. For strep-tagged LsrB, overexpression was induced using 200 ng/ml anhydrotetracycline in a 2 l culture volume, grown at 37°C for 3 h. Cells were pelleted by centrifugation at 11 000 × *g* for 15 min at 4°C, following lyses using the B-Per^Tm^ reagent (Thermo Fischer Scientific). Purification was performed according to the protocol and kit for “protein purification with Strep-Tactin^®^ resin” from IBA-Lifescience. Protein was eluted in buffer E (100 mM Tris–HCl, pH 8, 150 mM NaCl, 1 mM ethylenediaminetetraacetic acid, 2.5 mM desthiobiotin).

### Electrophoretic mobility shift assay (EMSA)

DNA-binding of LsrB was examined by electrophoretic mobility shift assay according to Müller *et al.* [52]. PCR-amplified promoter regions of target-genes were labeled with γ-^32^P-ATP. Free γ-^32^P-ATP was removed by gel-filtration (Probe Quant G-50 Micro Columns, GE Healthcare). Finally, labeled promoter regions were incubated with increasing concentrations of purified LsrB^His^ or ^His^ArgP for 20 min at 30°C and separated on a 6% polyacrylamide gel. Radioactive bands were documented by phosphoscreen exposure. For nonradioactive EMSAs (positive control for ArgP), 50 ng of PCR-amplified promoter regions of *lysP* [53] or *katG* were incubated with increasing amounts of purified ^His^ArgP in a final volume of 20 μl using binding buffer (10 mM Tris–HCl, pH 8.5, 10 mM MgCl_2_, 100 mM KCl, 0.1 mg/ml bovine serum albumin). After a 20-min incubation at room temperature, the reaction was stopped by adding 6 × Orange Gel Loading Dye (NEB). A total of 15 μl per sample was loaded onto a 2.5% agarose gel prepared with 0.5 × Tris-Borate-EDTA (TBE) buffer and electrophoresed at 120 V for 60 min. Gels were stained with GelRed (Biotium) at a 1:3000 dilution for 30 min and imaged using the ChemiDoc MP Imaging System (Bio-Rad).

### Susceptibility to ROS

*Agrobacterium tumefaciens* strains were cultivated overnight in LB medium and the optical density (OD_600_) was adjusted to 0.5 for spot assays or inhibition zone analysis. Inhibition zone analysis and spot assays were performed as previously described [37]. Here, 3 μl freshly prepared 1 or 2 M H_2_O_2_, or 0.2 M paraquat were added to the paper disks. Spot assays were performed on LB plates supplied with 1 mM H_2_O_2_, 0.1 mM paraquat, or 4 mM GSSG. ROS were added to 1.5% LB agar after autoclaving.

### Quantification of cell lengths

*Agrobacterium tumefaciens* strains were cultivated until mid-exponential phase and collected by centrifugation (11 000 × *g*, 1 min). The resulting pellets were washed with 1× PBS and adjusted to an OD_600nm_ of 0.5 in 1× PBS. Ten microliters of cell suspension was spotted on a microscopy slide coated with an agarose pad (1.5% w/v agarose in 1× PBS). Microscopy was carried using the Olympus BX51 fluorescence microscope at 100× magnification. ImageJ was used for image processing and cell lengths measurement.

### Seedling infection assay

T-DNA transfer capability of *A. tumefaciens* strains was analyzed by seedling infection assay using the AGROBEST method (*Agrobacterium*-mediated enhanced seedling transformation) as previously described [54, 55]. Here, 7-day-old *Arabidopsis thaliana efr-1* seedlings were infected with *A. tumefaciens* strains carrying the pBISN1 vector [56]; 3 dpi, seedlings were stained with X-Gluc (5-bromo-4-chloro-3-indolyl-β-D-glucuronide) overnight at 37°C.

### Motility assay

Cell-motility was examined on AB minimal medium (pH 5.5) supplemented with 0.375 % (w/v) agarose. Three microliters of overnight cultured cells was spotted on the plates. Swim rings were measured after incubation at 30°C for 48 h.

### Thiol-trapping and mass spectrometry

50 μg of protein (LsrB^Strep^) were tryptically digested using S-Trap micro spin columns (Protifi) following the manufacturer’s protocol. The reduction/alkylation step using tris(2-carboxyethyl)phosphine (TCEP) and methyl methanethiosulfonate (MMTS) was omitted for one part of the samples. Peptides were dissolved in 3% acetonitrile (ACN) and 0.1% formic acid (FA). For each sample, 4 μl of sample containing tryptic peptides of 0.25 μg of protein were subjected to high performance liquid chromatography (ACQUITY UPLC M-Class Chromatography System, Waters) using a nanoEase M/Z CSH130 column (300 Å, 1.7 μm, 300 μm × 100 mm; Waters); 0.1% FA in water (solvent A) or ACN (solvent B) were used for reverse phase chromatography. Peptides were eluted with a flow of 7 μl/min with the following gradient: initial, 1% B: 3 min, 1% B; 100 min, 35% B. Samples were eluted online to a Synapt XS mass spectrometer equipped with a ESI lockspray II source and a Low Flow ESI probe (Waters) using the following parameters: capillary voltage, 2.5 kV; Sampling Cone, 40; Source offset, 4; source temperature, 80°C; cone gas flow, 50 l/h; desolvation gas, 500 l/h; nebulizer, 3 bar. Continuous HDMS^E^ spectra were recorded in positive resolution mode with a mass range from 50 to 2000 Da and a scan time of 0.7 s. Collision energy was ramped from 17–60 V. [Glu1]-Fibrinopeptide B was measured as lock mass every 60 s.

Spectra were analyzed using the ProteinLynxGlobalServer (Waters, version 3.0.3) and an *E. coli* BL21 (DE3) database (Uniprot ID UP000002032) with manually added LsrB-Strep, trypsin, and keratin sequences with the following settings: chromatographic peak width, automatic; MS TOF (Mass Spectrometry-Time of Flight) resolution, automatic; lock mass for charge 2, 785.8426 Da/e; lock mass window, 0.25 Da; low energy threshold, 200 counts; elevated energy threshold, 25 counts; peptide tolerance, automatic; fragment tolerance, automatic; min fragment ion matches per peptide, 2; min fragment ion matches per protein, 5; min peptide matches per protein, 1; maximum protein mass, 250 000; primary digest reagent, trypsin; secondary digest reagent, none; missed cleavages, 3; fixed modifier reagents (if applicable), MMTS-alkylation C; variable modifier reagents, oxidation M, sulfenic acid C, sulfinic acid C, sulfonic acid C; false discovery rate, 4. Data are deposited at ProteomeXchange Consortium via the PRIDE repository with the identifier PXD055627.

### 4-acetamido-4′-maleimidylstilbene-2,2′-disulfonic acid trapping of free thiol groups

*In vitro* alkylation assays were performed with 17 μM purified LsrB^Strep^. A total of 100 μl purified LsrB was incubated with 100× molar excess of DTT (reducing) for 10 min at 25°C. Additives were removed by MicroSpin columns (Bio-Rad) and incubated with either 5 mM 4-acetamido-4′-maleimidylstilbene-2,2′-disulfonic acid (AMS) or water (control) for 30 min at 37°C. Samples were separated by nonreducing sodium dodecyl sulfate–polyacrylamide gel electrophoresis and visualized via Coomassie staining [57].

### Western blot

One microliter of cell culture was harvested by centrifugation (2 min at 16 000 × *g*). Pellets were resuspended in 1× protein sample buffer [2% (w/v) sodium dodecyl sulfate, 0.1% (w/v) bromophenol blue, 5% (w/v) β-mercaptoethanol, 10% (w/v) glycerol, 50 mM Tris–HCl, pH 6.8] according to their optical density, i.e. 100 μl per OD_600_ of 1. Samples were boiled at 95°C for 10 min before electrophoresis at 120 V. Western blot transfer was performed using the Bio-Rad Trans-Blot^®^Turbo^™^ system with 0.2 μm nitrocellulose membranes (Bio-Rad). Membranes were stained with Ponceau S [0.01% (w/v) Ponceau S, 1% (w/v) acetic acid] and documented in the ChemiDoc^™^ MP imager (Bio-Rad). Ponceau S-stained membranes were washed thoroughly with 1× PBST [10% (w/v) 10× PBS, 0.1% (w/v) Tween], followed by a 5-min blocking step in EveryBlot blocking buffer (Bio-Rad). For the detection of His-tagged proteins an anti-His-horseradish peroxidase (HRP) conjugated antibody (Qiagen, Cat. No. 34460) was used in a 1:4000 dilution. Chemiluminescence signals were detected using the ChemiDoc MP Imaging imager (Bio-Rad) by incubating the membranes with the Immobilon^®^ Forte Western HRP substrate (Merck).

### Structure prediction and structure modeling

Protein 3D models were generated using AlphaFold 3 [58]. AlphaFold 3 was run in standard settings and the top-ranking structure was used for further analysis. PyMol was used for visualization of the AlphaFold-generated models. The per-residue model confidence score (pLDDT) was visualized via the the PyMol extension https://github.com/cbalbin-bio/pymol-color-alphafold.

### Cryo-EM sample preparation

Quantifoil 1.2/1.3 200 mesh grids were glow discharged (Pelco EasiGlow) at 15 mA for 90 s before sample application. Three microliters of (1.2 mg/ml) sample was applied to the grid and vitrified with a Vitrobot (Thermo Fisher Scientific) at 4°C and 95% humidity. The grid was blotted for 7 s with a blot force of −5 before being plunge-frozen in liquid ethane.

### Cryo-EM data collection

For the single-particle analysis (SPA) of LsrB, 6216 movies were acquired with EPU using aberration-free image shift on a Titan Krios G4 electron microscope operating at 300 kV and a magnification of 105 000×. Data were recorded in counted super-resolution mode on a K3 BioContinuum detector, with a pixel size of 0.41 Å. Each movie comprised 60 frames, captured with a defocus range of −0.8 to −2.8 μm and a total electron dose of 60 e^−^/Å^2^.

### Cryo-EM data processing and model building

Movies were patch motion-corrected, CTF corrected and binned to physical pixel size of 0.82 Å in Warp [59]. Image processing was carried out in CryoSPARC (version 4.5.3 + 240 807) [60, 61]. Patch CTF-corrected images were used for particle picking with the blob picker tool, with a diameter of 50–200 Å. The particles were extracted with a box size of 180 pixels. 2D classification was used to select templates for template picking, which was performed with a diameter of 90 Å. A total of 13 366 702 particles were picked, of which 8864 865 were extracted with a box size of 200 pixels. Further 2D classification was performed, resulting in 4687 018 particles being used for two additional rounds of 2D classification, leading to a final particle stack of 1529 278 particles. *Ab initio* reconstruction was performed with three classes, of which one class containing 484 971 particles was used for 3D refinement with nonuniform refinement. This resulted in a final structure with a resolution of 3.9 Å.

An AlphaFold 3 model of the LsrB SBD dimer was used for rigid body fitting in UCSF ChimeraX [version 1.8 (2024–06-10)] [62]. The docked model was validated with Phenix (version 5419) [63].The cryo-EM structure data are deposited at the Protein Data Bank under the accession ID 9HH1.

### Statistical analysis

Statistical analysis was performed via GraphPad Prism 10. One-way ANOVA (Analysis of Variance) was employed to identify significant differences between the means of two or more independent groups.

## Results

### LsrB is a LTTR unique to Rhizobiales

**Figure 1.**
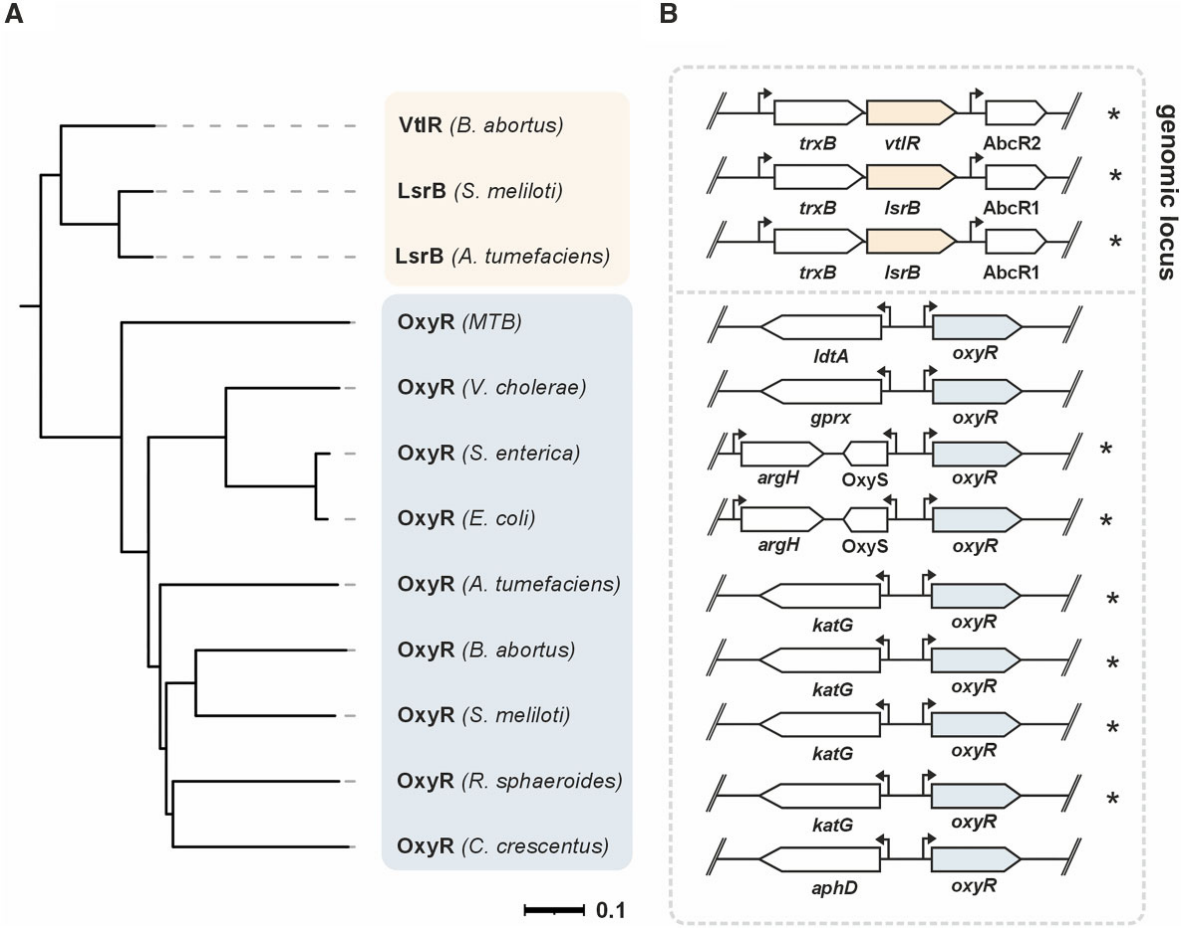
Phylogeny and genomic localization of the LTTRs LsrB and OxyR. (**A**) Homology tree of representative LsrB (Q2YRP4, A9CI74, Q92PZ7) and OxyR (L7N677, Q9KNU4, Q7CPB9, P0ACQ4, A9CGX9, Q2YK31, Q92RT2, Q3J2P7, Q9A269) proteins from selected alphaproteobacteria, gammaproteobacteria and actinobacteria. LsrB is unique to the alphaproteobacterial order Rhizobiales. Homology tree was calculated by Clustal Omega [69] and visualized via iTol [70]. Tree scale (genetic distance) = 0.1. MTB = *Mycobacterium tuberculosis*. (**B**) Genomic context of *lsrB* and *oxyR* in representative species as in panel (A). Conserved genomic contexts are highlighted by asterisks.

The concurrent presence of both OxyR and LsrB in Rhizobiales raises intriguing questions about the shared and specialized functions of these LTTRs. To explore their phylogenetic relationships and genomic contexts, we constructed a phylogenetic tree using representative LsrB and OxyR proteins from actinobacteria, gammaproteobacteria, and alphaproteobacteria. *A. tumefaciens, B. abortus*, and *S. meliloti* were chosen as representatives of pathogenic and symbiotic Rhizobiales, and *Caulobacter crescentus* and *Rhodobacter sphaeroies* represent free-living alphaproteobacteria (Fig. 1A). Phylogenetic analysis revealed that, after diverging from a common ancestor, two distinct lineages emerged, forming separate clusters for OxyR and LsrB. The lineage split suggests that OxyR and LsrB evolved to fulfill distinct functional roles. Interestingly, OxyR homologs were identified in all species analyzed, underscoring its critical role in oxidative stress protection. In contrast, the LTTR LsrB (referred to as VtlR in *B. abortus*) appears unique to Rhizobiales, suggesting a species-specific function for LsrB within this group (Fig. 1A).

Next, we examined the genomic contexts of *oxyR* and *lsrB* (Fig. 1B). Both genes are located adjacent to genes associated with oxidative protection. Notably, the genomic context of *oxyR* varies across species. In *E. coli* and *Salmonella enterica oxyR* is located upstream of the gene coding for the small RNA OxyS [64], while *Vibrio cholerae oxyR* is located adjacent to *gprx*, coding for a glutathione peroxiredoxin. In *Mycobacterium tuberculosis* (MTB), *oxyR* is adjacent to *ldtA*, which codes for a *L,D*-transpeptidase susceptible to oxidation [65]. In *C. crescentus*, *oxyR* is located upstream of *aphD*, coding for an alkyl hydroperoxidase reductase. Notably, despite their evolutionary distance, in both Rhizobiales and Rhodobacterales, *oxyR* was found upstream of *katG* (referred to as *katA* in some studies), coding for a bi-functional catalase-peroxidase, suggesting a functional link. For consistency in this manuscript, we will refer to the enzyme as KatG in accordance with the terminology in enterobacteria [28]. Previous research has shown that *katG* and *oxyR* are divergently transcribed from a bidirectional promoter in Rhizobiales, with *katG* transcription regulated by OxyR [24, 66].

Interestingly, in Rhizobiales *lsrB* consistently forms an operon with *trxB*, which encodes a thioredoxin reductase essential for maintaining cellular redox homeostasis by reducing oxidized thioredoxins using NADPH [21, 22, 67, 68]. In *A. tumefaciens*, this operon is subject to LsrB-dependent autoregulation [37].

### Single or double deletion of *lsrB* and *oxyR* effects ROS resistance, virulence, and motility

**Figure 2.**
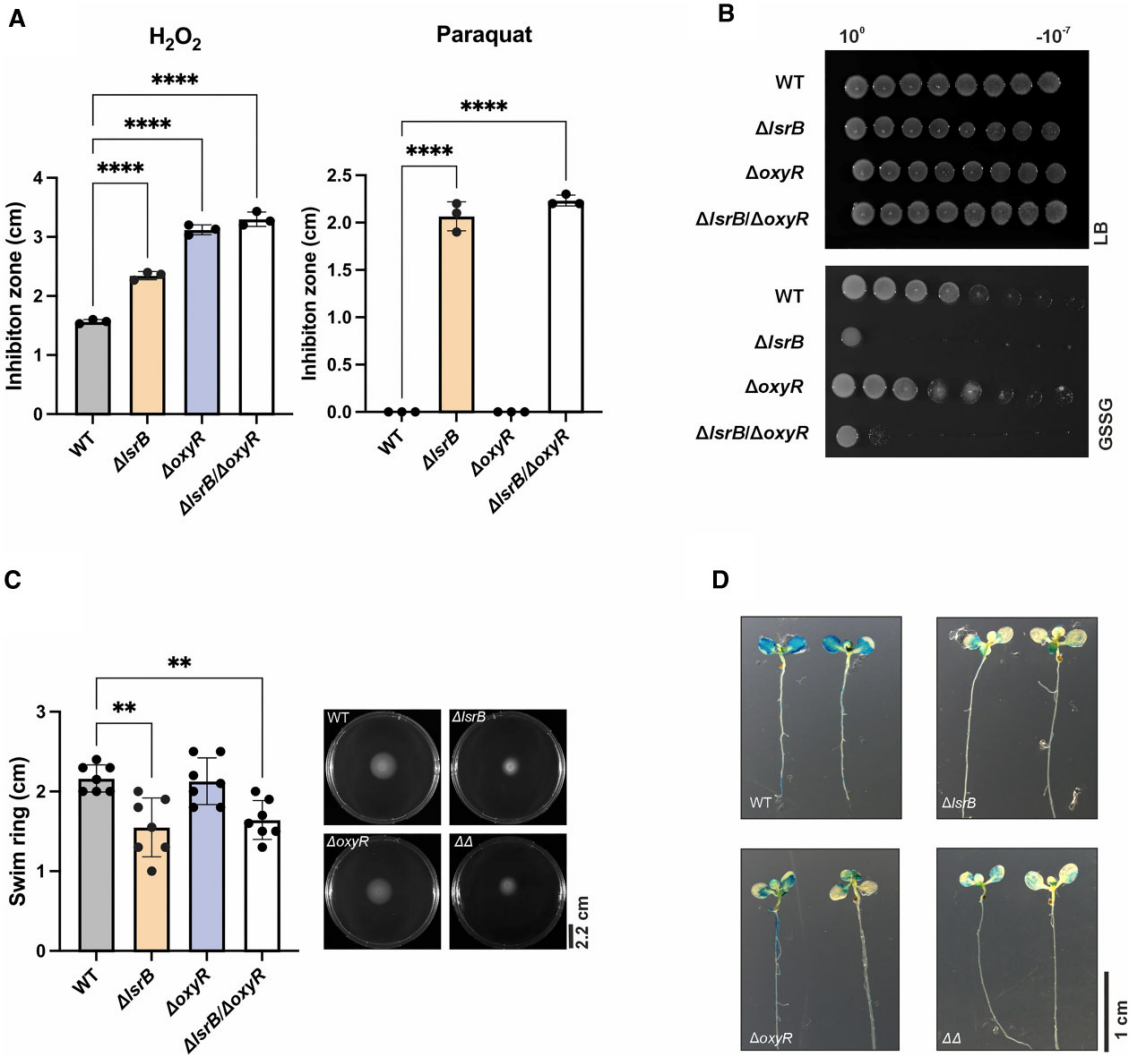
Phenotypic effects of *lsrB* and *oxyR* deletion. Susceptibility to ROS of *A. tumefaciens* WT, *ΔlsrB*, *ΔoxyR*, and *ΔlsrB/ΔoxyR* was examined by (**A**) Inhibition zone analysis. Inhibition zone diameters in the presence of 1 M H_2_O_2_ or 200 μM paraquat. Error bars represent ± mean standard deviation (SD) of three biological replicates. (**B**) Spot assay. Cells were cultivated overnight, adjusted to an OD_600nm_ of 0.5, serially diluted and spotted on LB plates supplied with the indicated stressor. Plates were documented after incubation at 30°C for 48 h. Images are representative for three independent replicates. (**C**) Cell motility analysis on AB, pH 5.5 soft-agar plates. *ΔΔ* = *ΔlsrB/ΔoxyR*. Error bars represent mean ± standard deviation of three biological replicates, with three technical replicates each. (**D**) Analysis of T-DNA transfer. *A. thaliana Δefr1* seedlings were infected with *A. tumefaciens* strains carrying the vector pB1SN1 (GUS-reporter gene [54]). Successful transfer of T-DNA (here *gusA*) results in β-glucuronidase expression in infected plant tissue. Cleavage of the substrate X-Gluc by the β-glucuronidase stains the respective tissue blue. Activity was measured 3 days dpi. Experiment was performed in three biological replicates, with 10 technical replicates each. Significance was tested by one-way ANOVA. *P < .05, **P < .01, ***P < .001, ****P < .0001.

To evaluate the phenotypic consequence of single or double deletion of *lsrB* and *oxyR* in *A. tumefaciens*, we first assessed the susceptibility to ROS. Compared to the WT, both single or double deletion of these regulators led to increased H_2_O_2_ sensitivity, as indicated by larger growth inhibition zones (Fig. 2A). This finding aligns with previous studies, reporting the H_2_O_2_ sensitivity of *lsrB* and *oxyR* single deletion mutants in Rhizobiales [24, 39, 66, 71]. Unlike the *ΔoxyR* mutant, the *A. tumefaciens ΔlsrB* and the *ΔlsrB/ΔoxyR* strains also showed sensitivity to paraquat, a bipyridinium herbicide that generates superoxide radicals, which are subsequently converted to destructive hydroxyl radicals [72].

We then tested sensitivity to GSSG by spot assays, building on prior findings of increased GSSG sensitivity of *S. meliloti ΔlsrB* [38, 39]. Consistent with the *S. meliloti* phenotype, both the *A. tumefaciens ΔlsrB* and *ΔlsrB/ΔoxyR* deletion mutants displayed increased sensitivity compared to WT and *ΔoxyR* (Fig. 2B). This elevated GSSG susceptibility might indicate an imbalance of the cytosolic glutathione homeostasis [73], suggesting that the redox buffer potential of glutathione is decreased in cells lacking LsrB. To address this, we determined the steady-state redox potential using the genetically encoded Grx1-roGFP2 probe that directly reflects the intracellular glutathione state [43]. Indeed, the redox potential exerted on Grx1-roGFP2 in the *lsrB* mutant compared to the WT was slightly shifted to more oxidizing conditions (Supplementary Fig. S1).

Next, we analyzed bacterial motility using a swim-ring assay on soft agar plates, following established protocols [74]. In line with previous findings [35], the *ΔlsrB* and the *ΔlsrB/ΔoxyR* mutant exhibited reduced motility, shown by smaller swim-ring diameters relative to WT and *ΔoxyR* (Fig. 2C). We also investigated whether deletion of the LTTRs affected cell morphology or length (Supplementary Fig. S2A). The changes were minor: cells of the *ΔlsrB* mutant were slightly elongated in comparison to the WT, whereas the *ΔoxyR* mutant cells appeared shorter.

Finally, to assess the role of these LTTRs in virulence, we performed a T-DNA transfer assay with *A. thaliana* seedlings [54, 55]. Blue staining of the infected plant tissue indicates successful T-DNA transfer, mediated by transient expression of the T-DNA-encoded *gusA* gene. Plants infected with *A. tumefaciens* WT carrying the infection vector exhibited strong GUS staining. However, single and double deletion strains drastically reduced T-DNA transfer efficiency, as evidenced by reduced GUS staining (Fig. 2D). The results corroborate previous observations of reduced tumor formation in the absence of *lsrB* [35, 36], emphasizing the critical roles of both LsrB and OxyR in *Agrobacterium* virulence.

### Deletion of *lsrB* and *oxyR* alters the transcriptomic response to hydrogen peroxide stress

**Figure 3.**
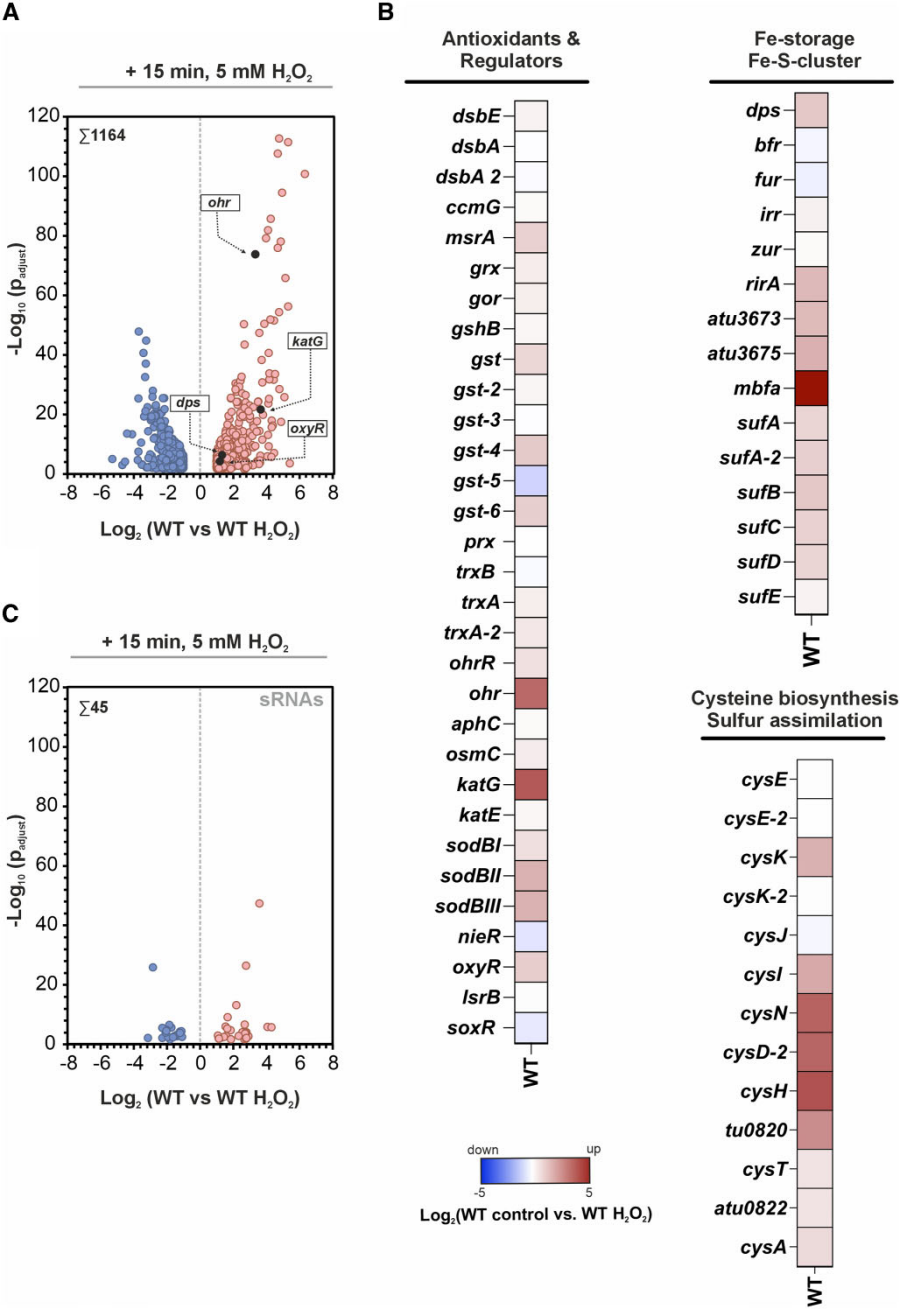
Transcriptomic response of *A. tumefaciens* WT to hydrogen peroxide stress. *Agrobacterium tumefaciens* WT strains were cultivated in LB medium until mid-exponential phase. Samples were withdrawn before (untreated) and after H_2_O_2_ treatment (“H_2_O_2_”) (**A**) Total number of differentially expressed genes in *A. tumefaciens* WT after hydrogen peroxide exposure (>2-fold, *P* < .01). Representative oxidative stress response genes are highlighted as dark-grey colored dots and labeled accordingly. (**B**) Heat map highlighting differentially regulated genes involved in the oxidative stress response (antioxidants, regulators, irons sequestration, or cysteine biosynthesis). (**C**) Total number of differentially regulated soluble RNA (sRNAs) in *A. tumefaciens* WT after H_2_O_2_ treatment.

To investigate the impact of single or double deletions of *lsrB* and *oxyR* on the transcriptomic response of *A. tumefaciens* to ROS, we performed RNA sequencing after a 15-min exposure to 5 mM H_2_O_2_ (Fig. 3 and Supplementary Table S4). The concentration of 5 mM H_2_O_2_ was chosen due to *A. tumefaciens*' relatively high peroxide tolerance. Concentrations of 20 mM or higher were reported as lethal, while low micromolar (μM) concentrations can induce protection against such high doses [66]. A previous study in the *A. tumefaciens* strain NL4 showed that gene expression changes already occur after 15 min of exposure to 0.25 mM H_2_O_2_ [24]. Based on these reports, our 15-min exposure to 5 mM H_2_O_2_ provides a suitable balance between inducing a clear oxidative stress response and minimizing cellular damage. Consistently, our pilot experiments revealed a clear transcriptional response after 15 min, with comparable *katG* induction at both low (0.1 mM) and intermediate (5 mM) H_2_O_2_ concentrations, suggesting robust activation of the oxidative stress response under our chosen conditions (Supplementary Fig. S2B).

For our global RNA sequencing approach, we subsequently considered genes showing at least a two-fold change in expression with a *P* < .01 were classified as differentially expressed (Fig. 3A). While collecting samples for RNA-seq, we quantified the colony-forming units (CFUs) following H_2_O_2_ treatment. The results show a significant reduction in CFU formation in the regulator mutants compared to WT (Supplementary Fig. S2C). Notably, the *ΔoxyR* and *ΔlsrB/ΔoxyR* strains exhibited the most pronounced decrease in survival. Although the survival rates dropped, viable cells were recovered for all strain.

In *A. tumefaciens* WT, exposure to H_2_O_2_ resulted in the differential expression of 1164 genes compared to the untreated control, indicating substantial transcriptomic remodeling (Fig. 3A). Key antioxidant genes (*sodBI, sodBII, sodBIII*, *katG*, *ohr*) were significantly upregulated, reflecting their central roles in oxidative stress protection in *A. tumefaciens* [66, 75–77] (Fig. 3B and Supplementary Table S4). Additionally, genes involved in iron storage (*dps*, *mbfA*, *rirA*), siderophore synthesis (*atu3673, atu3675*), iron-sulfur cluster assembly (*sufA-D*), cysteine biosynthesis, and sulfate assimilation/uptake (*cysI*, *cysK-2*, *cysN*, *cysD-2*, *cysH, atu0820*) were upregulated (Fig. 3B and Supplementary Table S4). This highlights the importance of (i) maintaining iron homeostasis to limit OH· radical generation through the Fenton reaction and (ii) supporting the renewal of oxidized cysteines [78–81].

Furthermore, the genes for the redox-responsive transcriptional regulators OhrR and OxyR were upregulated upon H_2_O_2_ treatment, aligning with their roles in activating antioxidant genes, such as *katG* and *ohr* in *A. tumefaciens* [66, 75, 82, 83] (Fig. 3A). Interestingly, we observed no differential expression in genes encoding thiol-disulfide interchange proteins (*dsbA, dsbE*, *ccmG)*, thioredoxins (*trx*), thioredoxin reductase (*trxB*), glutaredoxin (*grx*), the superoxide system regulator SoxR (*soxR*), or *katE*, coding for a monofunctional catalase (Fig. 3B and Supplementary Table S4). This result contrasts with *E. coli*, where genes for thioredoxins, glutaredoxin, and thiol-disulfide interchange proteins are upregulated under H_2_O_2_ exposure [18, 84]. These findings suggest that *A. tumefaciens* may utilize a distinct regulatory mechanism to maintain thiol-disulfide homeostasis. Notably, we identified 45 differentially regulated and largely uncharacterized sRNAs in response to H_2_O_2_ treatment, highlighting the potential role of sRNAs in the oxidative stress response of *A. tumefaciens* (Fig. 3C and Supplementary Table S4).

**Figure 4.**
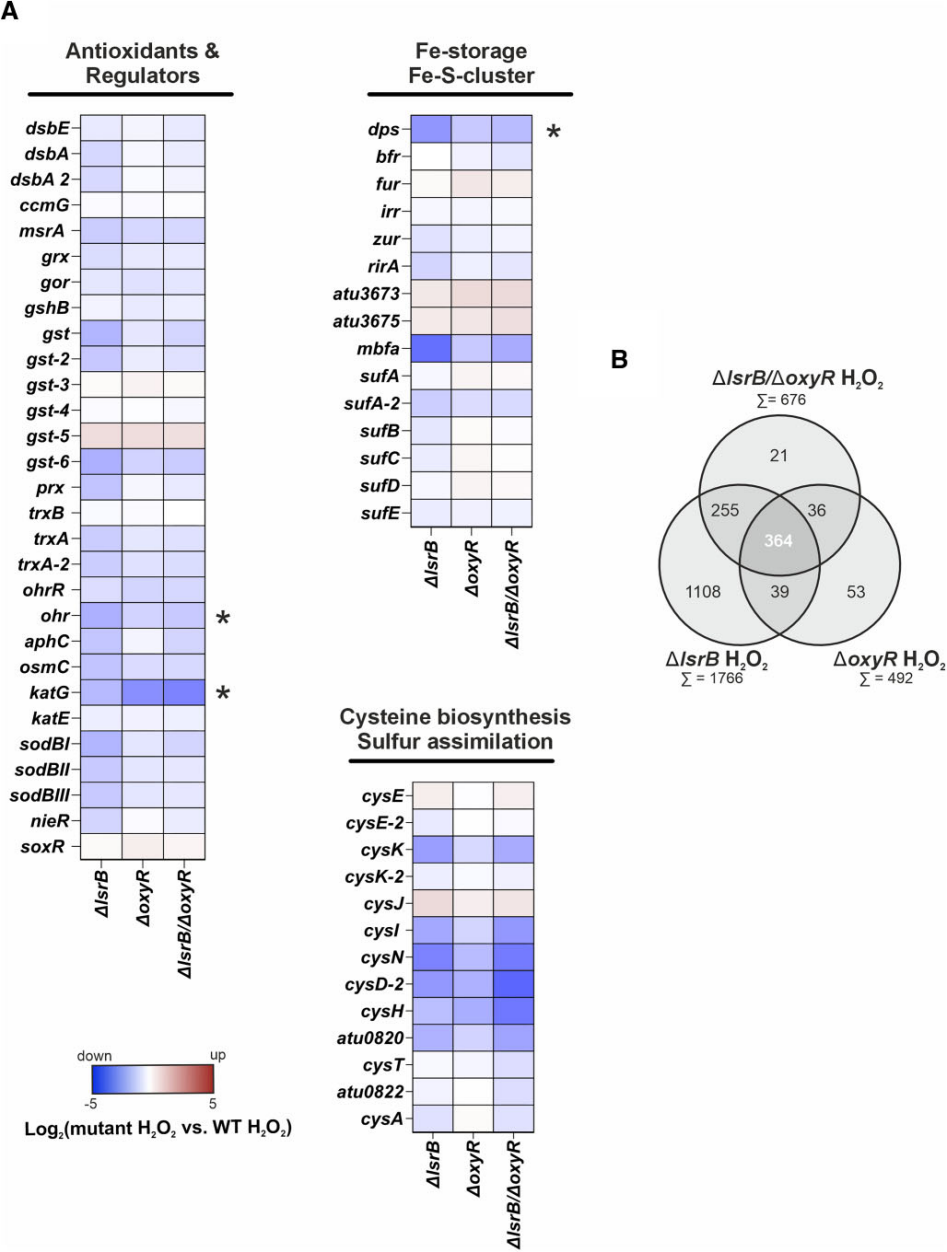
Effect of single or double deletion of *lsrB* and *oxyR* on the transcriptomic response to hydrogen peroxide stress. Strains were cultivated in LB medium until mid-exponential phase and collected before (untreated) and after H_2_O_2_ treatment (“H_2_O_2_”). (**A**) Heat map showing the expression of oxidative-stress associated genes in *A. tumefaciens ΔlsrB*, *ΔoxyR*, and *ΔlsrB/ΔoxyR* after H_2_O_2_ treatment. Expression of the regulator mutants is relative to H_2_O_2_-treated WT. (**B**) Comparison of the total number of differentially regulated genes between *ΔlsrB*, *ΔoxyR*, and *ΔlsrB/ΔoxyR* (>2-fold, *P* < .01). Asterisks highlight genes whose transcript levels were additionally examined by qRT-PCR (Supplementary Fig. S3 and Fig. 6).

Single and double deletions of *lsrB* and *oxyR* significantly altered the global transcriptomic response to H_2_O_2_ stress (Fig. 4 and Supplementary Table S4). In total, 1766 genes were affected by *lsrB* deletion, 492 by *oxyR* deletion, and 676 by *lsrB/oxyR* double deletion compared to WT after H_2_O_2_ treatment (Fig. 4B and Supplementary Table S4). Among these, 364 genes overlapped across the mutants, including those encoding antioxidant systems (*katG, ohr*), iron-sequestration (*dps*, *mbfA*), glutathione biosynthesis (*gst-6*), and cysteine biosynthesis/uptake (*cysI, cysN*, *cysD-2*, *cysH*, *atu0820*).

Interestingly, our data revealed a distinct OxyR regulon in *A. tumefaciens* (Fig. 4A). Genes typically regulated by OxyR in *E. coli* (e.g. *aphC*, *trxA*, *dsb*) were absent among the dysregulated genes [27, 31, 33, 66, 71, 85]. Instead, we identified genes associated with hydroperoxide response (*prx, osmC*), SOD (*sodBII*, *sodBIII*) thiol-homeostasis (*trxA*, *trxA-2*, *msrA*), iron sequestration (*bfr*), iron-sulfur cluster assembly (*sufA-2*), and glutathione-biosynthesis (*gst, gst-2*) uniquely downregulated in the *lsrB deletion* mutant. Additionally, *cysK* (cysteine biosynthesis), *aphC*, and *sodBI* were downregulated in both the *lsrB* and *lsrB/oxyR* double deletion mutants (Fig. 4A). Intriguingly, the redox-responsive transcription factors *nieR* (hypochlorite stress sensor), *rirA* (rhizobial iron regulator), and *ohrR* (hydroperoxide regulator) were downregulated in the *lsrB* mutant. Taken together, the transcriptomic response highlights the role of LsrB in the oxidative stress response.

### GO enrichment analysis of differentially regulated genes in response to hydrogen peroxide stress

**Figure 5.**
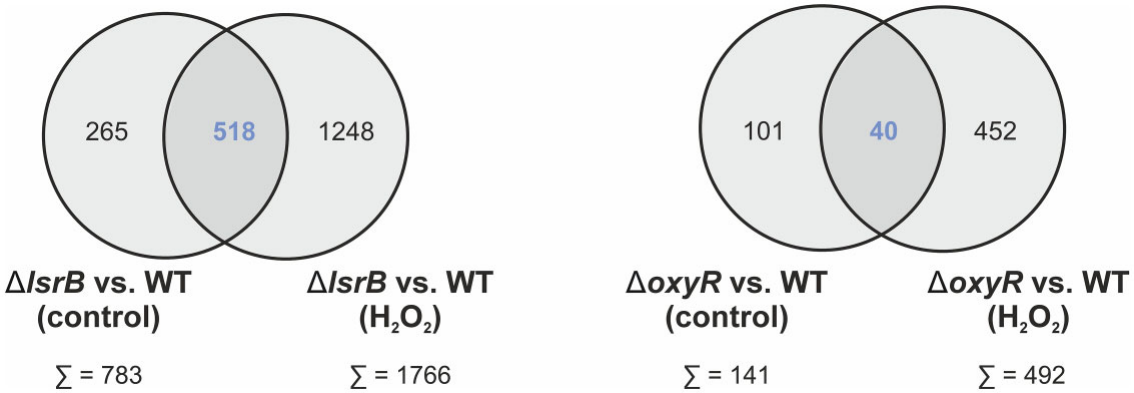
Overlap of genes affected by hydrogen peroxide stress in the absence of LsrB or OxyR. The Venn diagrams represent the total number of differentially regulated genes (>2-fold, *P* < .01) overlapping between *A. tumefaciens ΔlsrB* or *ΔoxyR* before and after H_2_O_2_ treatment.

To further analyze the oxidative stress-dependent regulons, we compared the number of differentially expressed genes in control conditions (LB medium) with those under oxidative stress in the single regulator mutants (Δ*lsrB*, Δ*oxyR*) (Fig. 5). The results show that the OxyR regulon is relatively small under control conditions, with 141 differentially expressed genes, while deletion of *lsrB* alone leads to the differential expression of 783 genes. Under oxidative stress, the number of differentially regulated genes expanded significantly to 1766 genes in the absence of *lsrB*, with 1248 unique to oxidative stress, and 492 genes in the absence of *oxyR*, with 452 unique to oxidative stress. This massive increase in differentially regulated genes highlights the substantial role of both regulators in managing oxidative stress responses, with *lsrB* potentially governing a broader stress-responsive network than *oxyR*. Consistent with previous studies reporting an extensive LsrB regulon, we found a substantial number of genes specifically regulated by LsrB [34–37].

To further refine the extensive set of differentially regulated genes, we performed a GO enrichment analysis. The overrepresented GO categories are listed in Supplementary Fig. S4A–C. We found that genes associated with translation (ribosomal RNA binding, ribosome), motility (flagellum), cell differentiation and chemotaxis were enriched in *A. tumefaciens* WT and the three regulator mutants (*ΔlsrB, ΔoxyR*, and *ΔlsrB/ΔoxyR*) upon H_2_O_2_ treatment. Notably, GO enrichments related to translation [transfer RNA (tRNA)], amino acid biosynthesis, ATP synthesis, and purine metabolism were absent in the mutants, indicating that regulation by LsrB and/or OxyR is crucial for these pathways (Supplementary Fig. S4A–C).

In contrast, we identified several GO terms uniquely enriched within the *lsrB* or o*xyR* deletion mutants, suggesting functional heterogeneity (Supplementary Fig. S4A–C). Consistent with previous studies [35, 36, 86], our GO enrichment analysis revealed that genes associated with transport (carbohydrates/transmembrane transport, monosaccharide binding, periplasmic space) and flagellar function were enriched in *ΔlsrB*. This enrichment likely accounts for the observed motility defect of *ΔlsrB*. In the *oxyR* and *lsrB*/*oxyR* deletion mutants, we exclusively found enrichments of genes associated with glyoxylate and glycolate catabolism, oxidative phosphorylation, protein unfolding, chaperone activity, DNA repair, ubiquinone biosynthesis, and sulfate assimilation. This suggests an increased demand for electron transport chain activity, potentially as a compensatory response to maintain cellular energy production. Additionally, the enrichment of genes related to protein unfolding and chaperone activity underscores the need for effective protein quality control.

Surprisingly, we identified unique enrichment of genes associated with ribose catabolism and monoatomic ion transport in the *ΔlsrB/ΔoxyR*. We propose that ribose may be utilized as an alternative energy source or that the oxidation of ribose moieties in nucleic acids necessitates rapid renewal. The enhanced monoatomic ion transport could indicate an increased demand for ion homeostasis in the double mutant.

In summary, we found that the response of *A. tumefaciens* to oxidative stress is characterized by the enrichment of genes related to translation, amino acid biosynthesis, ATP synthesis, purine metabolism, motility, cell differentiation, and chemotaxis. This indicates a robust and coordinated effort to sustain cellular function and growth. Furthermore, our findings underscore the distinct regulatory roles of LsrB and OxyR during oxidative stress. The unique and shared gene enrichments suggest complex regulatory networks that maintain cellular function and energy homeostasis.

### Target validation shows that *katG* is directly regulated by both OxyR and LsrB

**Figure 6.**
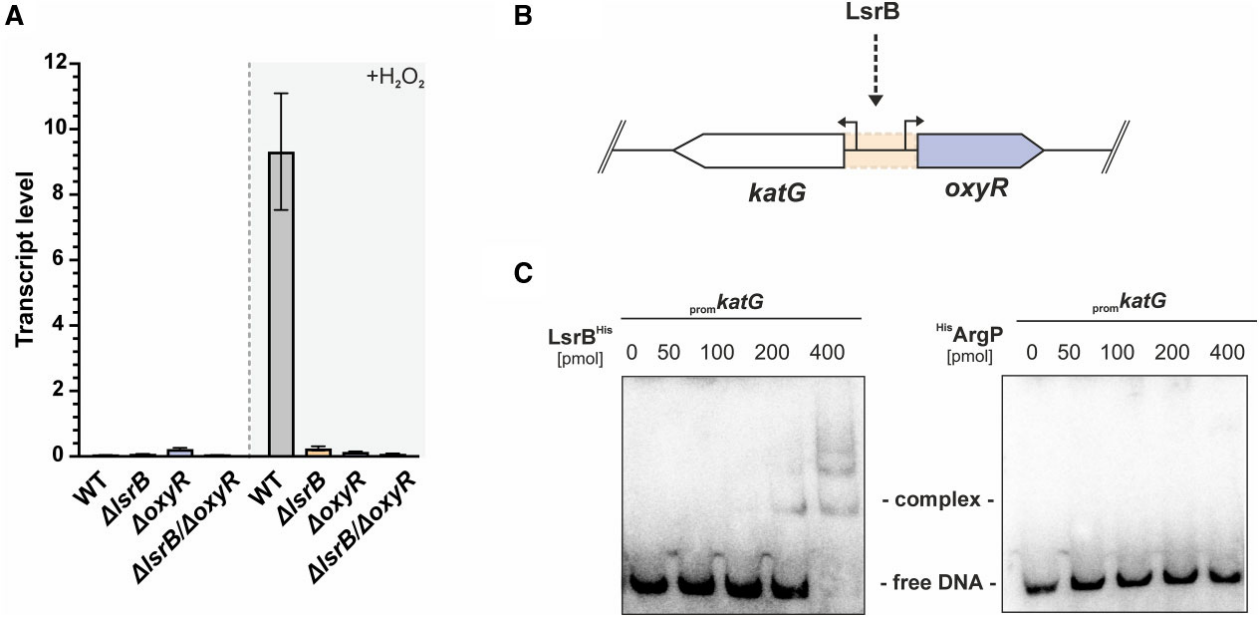
LsrB is the direct regulator of *katG* and *oxyR*. (**A**) Transcript levels of *katG* (*atu4642*) from cells grown in the LB medium to mid-exponential phase. Samples were collected before or after 15 min, 5 mM H_2_O_2_ exposure. Transcript levels were normalized to *gyrB*-levels. Error bars represent ± standard error of the mean of three independent replicates. (**B**) Binding of LsrB within the intergenic region of *oxyR* and *katG*, highlighted in orange. (**C**) DNA–protein interactions. A 125 bp fragment upstream of the *katG* CDS was labeled with ^32^P and incubated with increasing concentrations recombinantly purified LsrB^His^ or ^His^ArgP (nonbinding control). Herring sperm was utilized as competitor DNA. The protein–DNA complex and free promoter DNA are labeled. Band shift assay was performed in three independent replicates, with one representative shown here.

To validate the differential regulation of key antioxidant genes, we performed qRT-PCR on selected genes (*dps*, *ohr*, *katG*) under oxidative stress induced by H_2_O_2_. The results confirmed that both OxyR and LsrB positively regulate *katG* and *dps* expression under oxidative stress because induction of these transcripts was not observed in the LTTR mutants in the presence of H_2_O_2_ (Fig. 6A and Supplementary Fig. S3). In case of *ohr*, only LsrB appeared to be a positive regulator (Supplementary Fig. S3B), which contrasts with the RNA-seq results, where also OxyR was found to regulate *ohr* expression albeit not as much as LsrB. This discrepancy could be attributed to differences in experimental conditions, differences in detection sensitivity between qRT-PCR and RNA-seq, or the presence of additional regulatory layers not captured by RNA-seq. To examine potential cross-regulation of the two LTTRs, we measured *lsrB* and *oxyR* expression in the respective mutant strains and found that *oxyR* transcripts are not upregulated in the absence of LsrB (Supplementary Fig. S3C and D).

The bi-functional catalase-peroxidase KatG is one of the best-characterized positively regulated targets of OxyR in *A. tumefaciens* [24, 66]. Our RNA-seq and qRT-PCR results uncovered LsrB as an additional regulator of *katG* and as potential regulator of *oxyR*. Therefore, we asked whether LsrB directly binds to the intergenic region between *katG* and *oxyR* (Fig. 6B) and conducted EMSAs using the radio-labeled promoter region of *katG/oxyR*. Increasing concentrations of purified LsrB^His^ or ^His^ArgP were incubated with promoter region (Fig. 6C). The presence of a distinct band shift indicates that LsrB directly binds to the region between *katG* and *oxyR*, which further supports a direct role of LsrB as a positive regulator under oxidative stress. To further validate the specificity of our observed binding, we performed a band shift assay including two controls: a nonbinding control for LsrB (*ampC*, as previously published [37]; Supplementary Fig. S5A) and a positive control for ArgP, demonstrating its binding to its target promoter, *lysP* (Supplementary Fig. S5B), as previously reported [53].

### Ectopic *katG* expression recovers the hydrogen peroxide sensitivity of *lsrB* and *oxyR* deletion mutants

**Figure 7.**
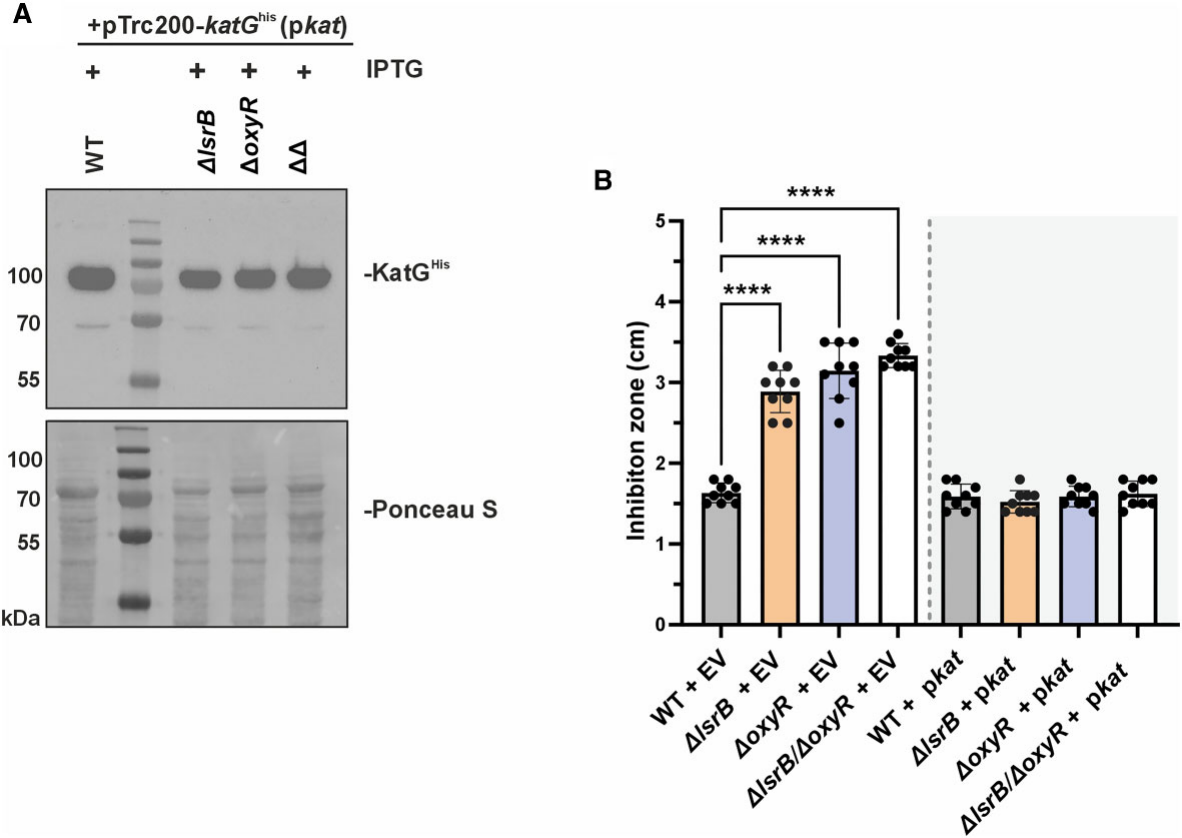
Overexpression of *katG* in Δ*lsrB* and Δ*oxyR* mutants restores resistance to H_2_O_2_. (**A**) Western blot detection of plasmid-derived KatG^His^ in *A. tumefaciens* WT, Δ*lsrB*, Δ*oxyR*, and Δ*lsrB/*Δ*oxyR* strains. (**B**) Inhibition zone analysis in the presence of 1 M H_2_O_2_ after incubation at 30°C overnight. Error bars represent ± mean SD of three biological replicates. ΔΔ = *ΔlsrB/ΔoxyR*; EV = empty vector (pTrc200).

KatG is the primary enzyme responsible for H_2_O_2_ detoxification in *A. tumefaciens* [24, 66, 76]. Based on this, we hypothesized that introducing plasmid-encoded *katG*, under the control of an IPTG-inducible promoter, might cure the H_2_O_2_ sensitivity of the *oxyR* and *lrsB* mutants to WT levels. We confirmed KatG (with a His tag) production through western blot analysis (Fig. 7A). Inhibition zone assays in the presence of H_2_O_2_ showed that “boosted” *katG* expression complemented the H_2_O_2_ sensitivity of the *ΔlsrB* and *ΔlsrB/ΔoxyR* mutants and restored WT-like resistance (Fig. 7B, compare with Fig. 2A). Overall, these findings highlight the critical role of KatG in H_2_O_2_ defense and further support the function of LsrB and OxyR as direct regulators of *katG*.

### Does LsrB directly sense ROS?

OxyR is a well-characterized thiol-based redox switch [29, 87, 88]. Cumulatively, the data presented above suggested that LsrB shares key ROS-response targets with OxyR under H_2_O_2_ stress, which led us to investigate whether and how LsrB directly senses ROS.

**Figure 8.**
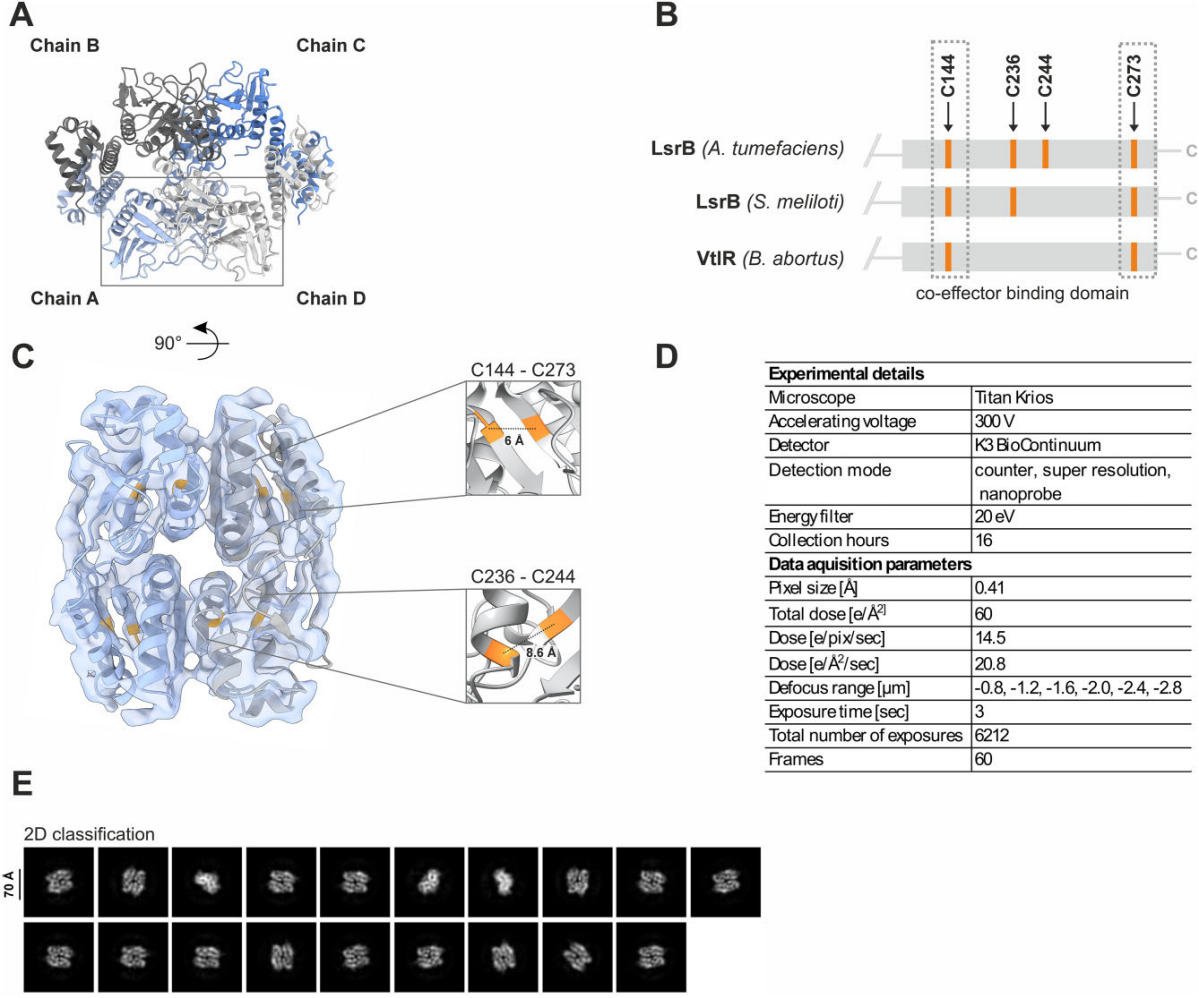


Using AlphaFold 3 [58], we obtained a structural model of the LsrB homotetramer (Fig. 8A and Supplementary Fig. S6B). The model indicates that LsrB monomers interact through their co-effector binding domains, a mechanism well documented for several LTTRs, such as CbnR [89, 90]. The C-terminal co-effector binding domain harbors four cysteine residues (Fig. 8B). Sequence comparison with LsrB homologs from *S. meliloti* and *B. abortus*, which only contain three or two cysteines, respectively, revealed that residues C144 and C273 are conserved (Fig. 8B and Supplementary Fig. S6A).

To experimentally support the biocomputational structure model, we employed cryo-EM of purified LsrB. Instead of the expected tetramers, we captured LsrB dimers, likely due to unstable homotetrameric oligomerization in the absence of DNA (Fig. 8C–E). We excluded the DNA-binding domain from our analysis due to its high flexibility, prior to refining the volume density map (resolution 3.9 Å) and fitting it with the AlphaFold 3-generated model (Fig. 8C and Supplementary Fig. S7). The results show that AlphaFold accurately predicted both (i) the dimeric interface of the two LsrB monomers and (ii) the spatial arrangement of the chains.

Based on the validated 3D structure, we were able to localize the two pairs of cysteine residues (C144/C273 and C236/C244; Fig. 8C). Unfortunately, AlphaFold 3 does not distinguish between reduced and oxidized conformations. We calculated the distances between the cysteine residues in each pair and found them to be below 10 Å (Fig. 8C). We suspect the residues to be in closer proximity in the true oxidized form, as several structural studies on the LTTR OxyR have revealed variable distances between its redox-active cysteines, C199 and C208 [28, 29, 87, 88]. In the reduced OxyR conformation, these residues are separated by 17 Å, but their oxidation leads to a large conformational change that brings them together [88].

**Figure 9.**
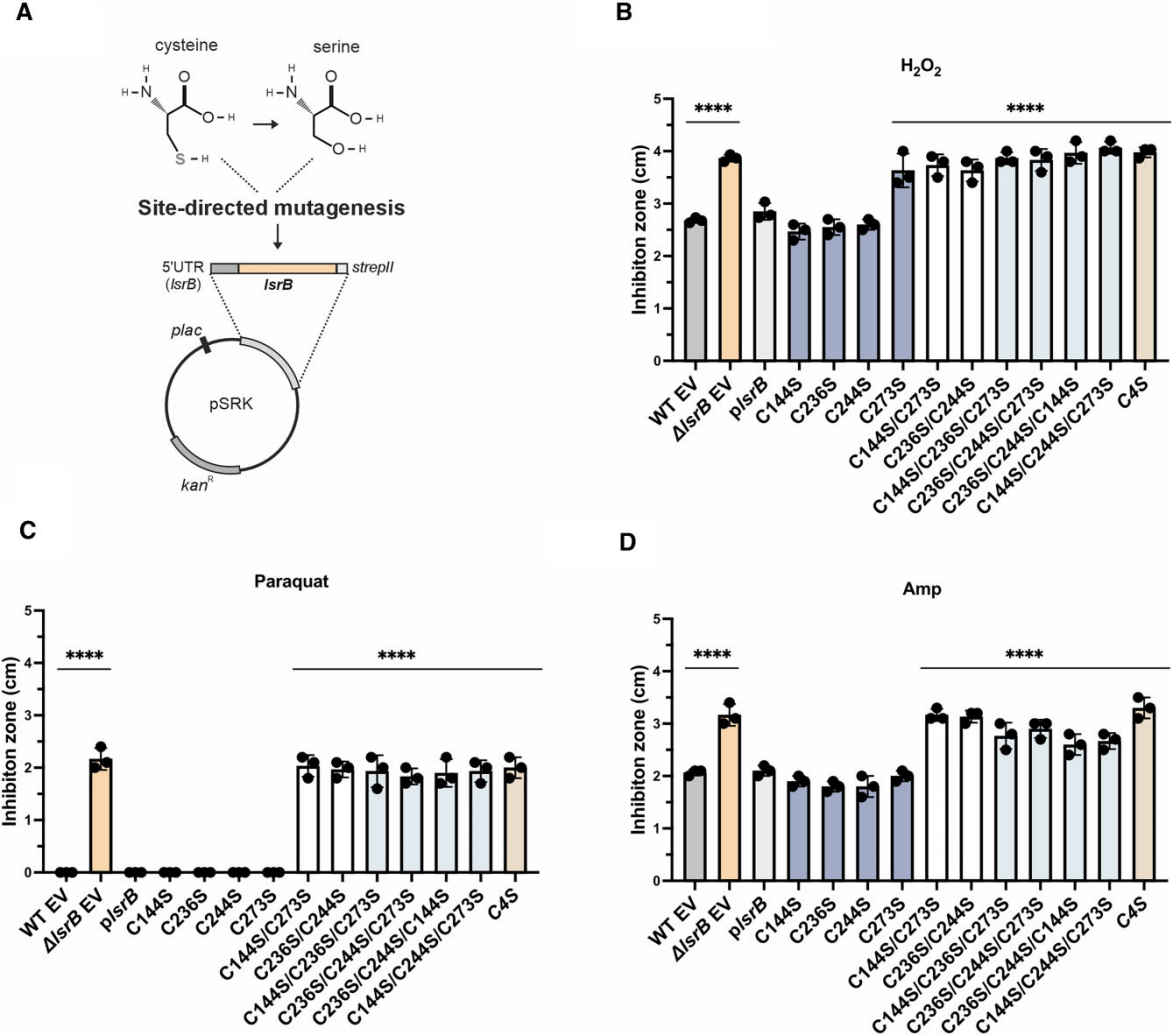
Phenotypic consequences of cysteine residue substitutions in LsrB. (**A**) Substitution of cysteine to serine was performed via site-directed mutagenesis on the *lsrB*-complementation plasmid. The resulting variants where phenotypically characterized by inhibition zone analysis in the presence of (**B**) 2 M H_2_O_2_, (**C**) 0.2 μM paraquat or (**D**) 100 mg ml ampicillin [37]. Error bars represent ± mean SD of three biological replicates. Significance was tested by one-way ANOVA (versus WT-EV). *P < .05, **P < .01, ***P < .001, ****P < .0001. EV = empty vector (pSRK).

Next, we experimentally examined the role of the cysteine residues in *A. tumefaciens* LsrB by (i) performing mutagenesis studies *in vivo* and (ii) using an LC/MS (Liquid Chromatography-Mass Spectrometry) based thiol-trapping approach to analyze cysteine modification *in vitro*. We used the *lsrB-*complementation plasmid [36] to substitute cysteine residues with Ser, as the two amino acids are structurally similar except for the redox-active thiol group (Fig. 9A). Substitution of the nonconserved cysteine residues (C236, C244) and the conserved C144 with Ser did not affect LsrB function, indicated by WT-like sensitivity to ROS or ampicillin (Fig. 9B and C). In contrast, substitution of C273 led to increased H_2_O_2_ sensitivity reminiscent of the *lsrB*-deletion mutant phenotype (Fig. 9B). This suggests that oxidation of C273, i.e. to sulfenic acid could be sufficient to modulate LsrB activity in the presence of H_2_O_2_. This aligns with findings in *E. coli* OxyR, where substitution of the redox-active C199 with Ser impaired the regulator’s DNA-binding ability [91]. Additionally, the C199S mutation specifically inhibited OxyS transcription [87]. Interestingly, the C273S exchange in LsrB had no effect on the paraquat and ampicillin sensitivity (Fig. 9C and D). All cumulative mutations by substitution of the putative redox-active cysteine pairs (C144S/C273S; C236S/C244S) as well as triple and quadruple substitutions led to phenotypes as in the *lsrB* deletion strain (Fig. 9B and C).

To confirm redox sensing by LsrB, we used two different biochemical assays. First, we conducted conventional AMS trapping. Purified LsrB was reduced using DTT and subsequently alkylated with AMS (Supplementary Fig. S8). A small upshift of the protein band from the control (water) sample to the DTT-treated sample suggests alkylation of free thiols.

**Figure 10.**
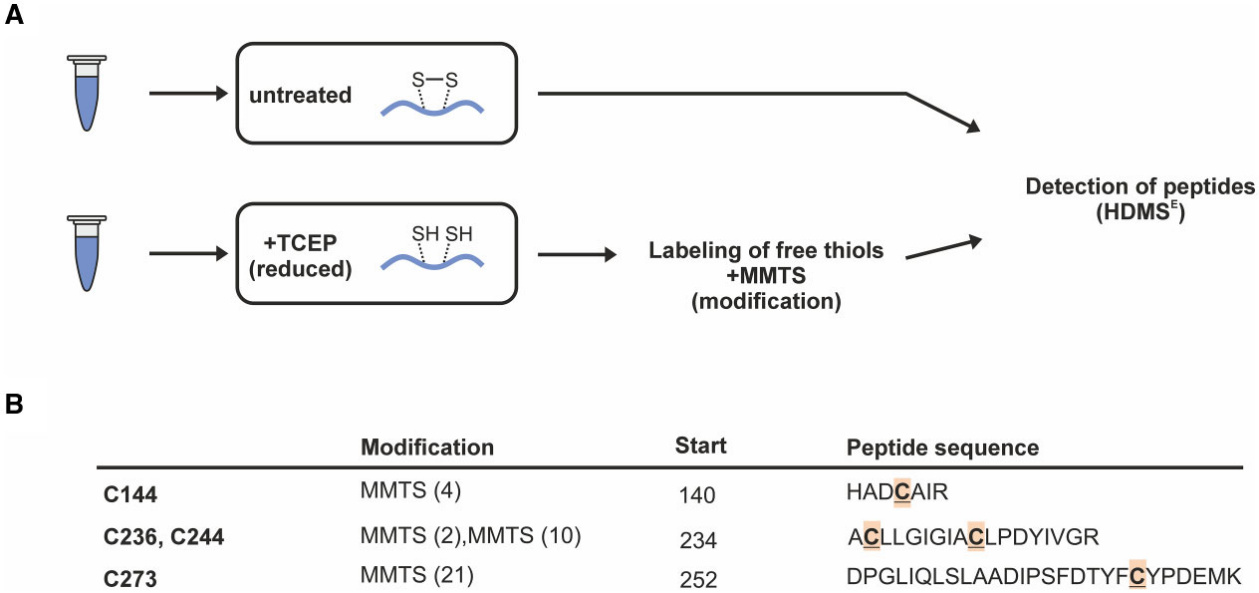
Thiol-proteomics. (**A**) Recombinantly purified LsrB^Strep^ was either left untreated for tryptic digest or was subjected to reduction via TCEP and modification of free thiols by MMTS. Peptides were detected via HDMS^E^ (data-independent high-definition mass spectrometry). (**B**) Summary of detected MMTS modification after TCEP reduction. Peptide sequence and positions are given ibid. Experiments were performed in two independent replicates.

We then used mass spectrometry to assess if the cysteines of recombinantly purified LsrB can be monitored by bottom-up proteomics (Fig. 10A). To this end, the protein was first tryptically digested and analyzed by mass spectrometry without modification of cysteine residues. We did not identify peptides containing the cysteine residues in the untreated sample, likely due to the formation of disulfide linkages or modifications not present in the database, which would render them undetectable. When LsrB was reduced with TCEP, which breaks the S-S bond and converts the cysteine residues back to their reduced form (-SH) [92], and subsequently subjected to modification of cysteines with MMTS, which “traps” free thiol groups by forming mixed disulfides [93], peptides containing all four cysteine residues (C144, C236, C244, C273) were identified with MMTS modification (Fig. 10B). This result strongly suggests that all four residues undergo reversible thiol modification, indicated by the subsequent MMTS modification in the reduced sample. Based on the protein structure (Fig. 8), we propose that the conserved residue C273 forms a disulfide bond with the proximal thiol C144, while the two nonconserved residues, C236 and C244, form another disulfide linkage.

## Discussion

ROS are an unavoidable consequence of life in oxygen-rich environments and are released by eukaryotic cells during bacterial infections. ROS are powerful agents that can damage DNA, proteins, and lipids, making it essential for bacteria to mount effective defense strategies [10]. The primary mechanisms for counteracting oxidative stress involve antioxidant systems (ROS detoxifying enzymes), including SOD, peroxiredoxins, and catalases [1, 11]. These systems are often regulated by ROS-sensing transcriptional regulators, such as the LTTR OxyR. The existence of LsrB, another putative redox-sensing LTTR in *A. tumefaciens*, raises questions about how OxyR and LsrB coordinate the oxidative stress response in this plant pathogen. Our findings suggest a division of labor between these proteins: while OxyR primary mitigates hydrogen peroxide stress, LsrB plays a broader function in protecting against diverse environmental stressors, including, but not restricted to oxidative stress.

### LsrB acts as redox sensor

A significant finding of our study is that LsrB functions as thiol-based redox switch. With four cysteines in its co-effector binding domain, LsrB is ideally structured to build two reversible disulfide bonds in response to oxidative state of the cytoplasm. Like the paradigmatic example OxyR, the reversible nature of thiol modifications enables LsrB to switch between oxidized and reduced states [18, 19]. Contrary to a previous report suggesting that *S. meliloti* LsrB forms intermolecular disulfide bonds between cysteines from different monomers [39], our findings support a more plausible alternative. The cryo-EM structure, combined with structural modeling, provides compelling evidence for the formation of intramolecular disulfide bonds involving both conserved (C144–C273) and nonconserved (C236–C244) cysteine residues (Fig. 8). Additionally, the predicted spatial separation of over 50 Å between cysteine residues from different monomers strongly argues against the possibility of intermolecular disulfide bond formation.

Further evidence supporting *A. tumefaciens* LsrB’s ability to sense the redox state through reversible disulfide bond formation includes the following observations: (i) substitution of C273 with serine, either alone or in combination with other cysteine to serine exchanges, resulted in increased sensitivity to H_2_O_2_, and (ii) *in vitro* thiol trapping showed that all four cysteine residues in LsrB undergo reversible thiol-based modifications. A central role for cysteine residues in the functionality of LsrB is in agreement with mutagenesis studies on *S. meliloti* LsrB, which contains three cysteine residues [39].

The exact roles of the two cysteine pairs in *A. tumefaciens* LsrB, particularly the nonconserved residues (C236 and C244), remain to be elucidated. A possible explanation could be a redundant function of the conserved (C144 and C273) and nonconserved (C236 and C244) cysteine pairs. Determining whether they serve regulatory or auxiliary functions will require optimized thiol-trapping approaches to better resolve their oxidation and reduction dynamics with greater spatial and temporal precision. In contrast, *E. coli* OxyR presents a simpler model system, as the protein contains one cysteine pair and its oxidation and reduction kinetics have been well-characterized [28, 94].

**Figure 11.**
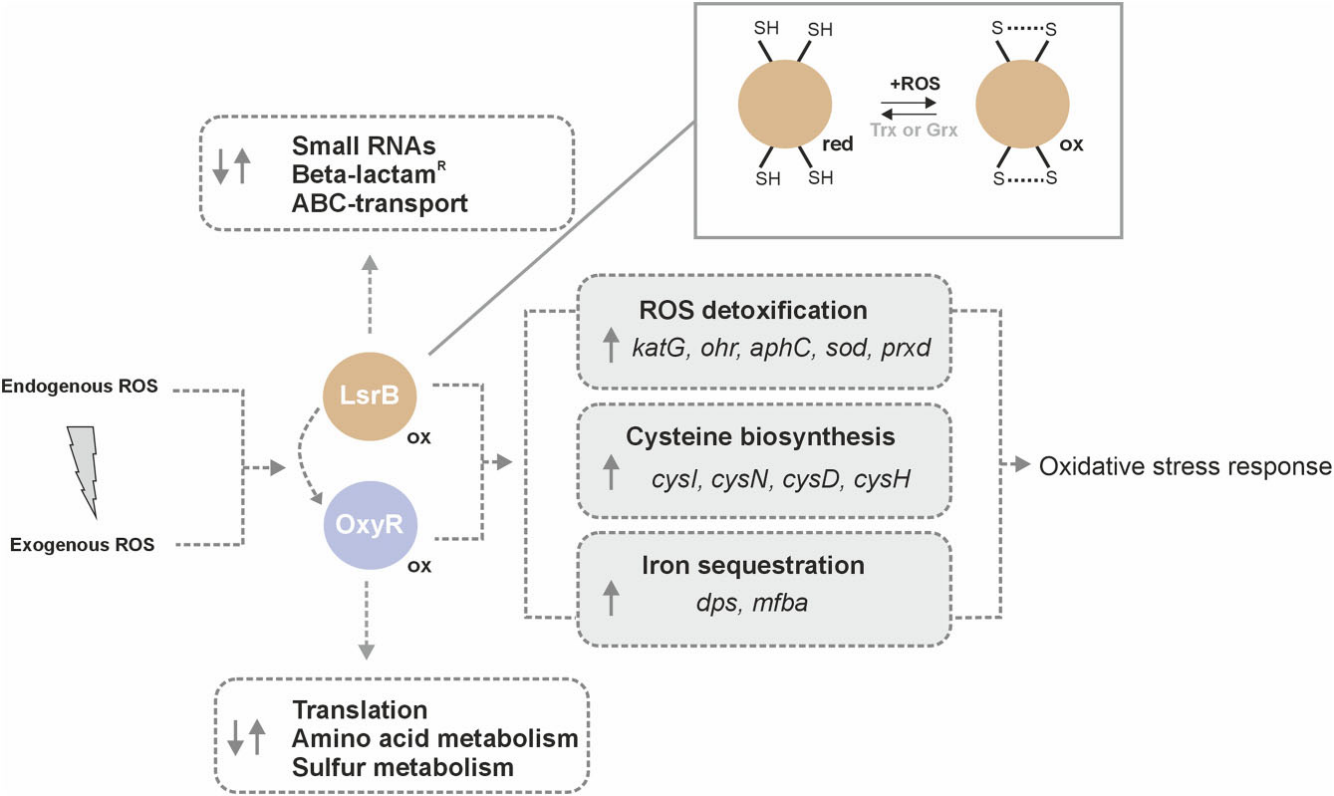
Regulation of the oxidative stress response in *A. tumefaciens* by two LTTRs. In response to ROS, the LTTRs LsrB and OxyR activate the transcription of key-antioxidant systems, ferritins, and cysteine biosynthesis. While the mechanism of OxyR as thiol-based redox sensor is well documented, our study uncovered that *Agrobacterium* LsrB acts a thiol-based redox switch, by utilizing redox-active cysteine residues for activation and is eventually re-reduced by the thioredoxin (Trx) or glutaredoxin system (Grx).

Notably, in Rhizobiales, the *lsrB* gene is consistently co-localized in an operon with *trxB* (Fig. 1), which encodes a thioredoxin reductase, suggesting that the thioredoxin system (TrxA, TrxB, NADPH) may play a role in the re-reduction of LsrB (Fig. 11). However, testing this hypothesis has proven challenging due to the essential role of the Trx system (*trxA*, *trxA-1*, *trxB*) in maintaining thiol-redox homeostasis [13, 21]. Despite multiple attempts, we have been unable to construct a viable *trxB* mutant.

### How *Agrobacterium* responds to hydrogen peroxide stress

Our study provides the first comprehensive insight into the transcriptomic response of *Agrobacterium* to H_2_O_2_ stress. Despite large variations in experimental approaches in previous studies from the last 20 years on free-living and host-associated alphaproteobacteria [33, 95–97], both the published and our results consistently show an upregulation of antioxidants systems (primarily catalases) and iron sequestration. In *Agrobacterium*’s close relative, *S. meliloti*, exposure to 0.5 mM H_2_O_2_ for 30 min resulted in the upregulation of key oxidative stress genes, including *katG* and *oxyR* [97]. In *A tumefaciens* we found upregulation of *katG* and *oxyR* 15 min after H_2_O_2_ exposure (Figs 3 and 4 and Supplementary Table S4). Notably, our pilot experiments assessing suitable time points and concentrations for transcriptomic analysis showed that *katG* expression, used as a proxy for oxidative stress response, changed as early as 5 min, with a stronger induction at 15 min (Supplementary Fig. S2B). A prolonged activation of oxidative stress response genes in host-associated species may reflect an adaptation to persistent oxidative challenges in the host environment.

Like many other bacteria, *Agrobacterium* responds to H_2_O_2_ stress by activating antioxidant systems, ferritin production, and amino acid biosynthesis to facilitate rapid ROS decomposition and repair of damaged proteins [95, 97, 98]. The differential regulation of genes related to translation and nucleotide biosynthesis suggests that *Agrobacterium* prioritizes stress responses and repair pathways over growth to counteract oxidative stress, a widespread adaptive strategy observed across various bacterial species [1, 12, 84, 97].

Unexpectedly, *ohr*, which encodes a hydroperoxide resistance protein involved in organic oxidant defense, was upregulated under our experimental conditions. This contrasts with previous findings indicating no induction at lower H_2_O_2_ concentrations (200 μM) [75]. This discrepancy may be attributed to *A. tumefaciens*’ ability to rapidly detoxify low H_2_O_2_ levels. We propose that the higher H_2_O_2_ concentration (5 mM) used in our study led to an increased generation of •OH via the Fenton reaction, which could also account for the observed upregulation of ferritin genes. The •OH radicals likely triggered the formation of organic hydroperoxides, such as lipid hydroperoxide (LOOH), through lipid peroxidation [99]. This would explain the increased requirement for Ohr to decompose the organic hydroperoxides, aligning with observations in other Gram-negative bacteria [82, 99, 100].

In response to H_2_O_2_ exposure, *A. tumefaciens* also upregulated cysteine biosynthesis, a response resembling the CysB-mediated pathway in *E. coli* [84]. Interestingly, a BLASTp search identified only a distantly related LTTR protein (Atu3381) with 24.7% homology to CysB. Based on our transcriptomic studies, Atu3381 does not appear to respond to oxidative stress. This lack of responsiveness, combined with its low sequence similarity to CysB, suggests that in *A. tumefaciens*, the roles of LsrB and OxyR may have evolved to regulate the *cys* genes. This potentially compensates for the function typically managed by CysB in other bacteria.

### OxyR and LsrB co-regulate central oxidative stress response pathways

Our findings suggest that a functional interplay between LsrB and OxyR is essential for the defense of *A. tumefaciens* against oxidative stress. Both deletion mutants of *lsrB* and *oxyR* mutants were highly sensitive to H_2_O_2_. All mutants exhibited reduced virulence, likely due to their inability to counteract the plant’s oxidative burst effectively [101]. We conclude that these phenotypic changes result from impaired upregulation of key antioxidant systems. LsrB and OxyR are required for activating genes involved in antioxidant defense (k*atG*, *ohr*), cysteine biosynthesis, and iron sequestration (*dps*) in response to H_2_O_2_ (Fig. 11).

Interestingly, *katG* and *dps* were previously identified as part of the core OxyR regulon (reviewed in [18]), suggesting a convergence between OxyR and LsrB pathways under oxidative stress. However, many genes dysregulated in the *lsrB* mutant remained unaffected in the double deletion mutant, implying that the simultaneous deletion of both regulators introduces unknown synergistic effects that significantly alter the transcriptome. Surprisingly, we observed that the *lsrB* and *lsrB/oxyR* double deletion mutants are also sensitive to GSSG and paraquat, suggesting that LsrB responds to a broader spectrum of oxidative stress signals beyond H_2_O_2_. Increased levels of paraquat and GSSG disrupt the cellular redox balance [72, 73], indicating that LsrB may have a more expansive role in redox regulation.

### Fail-safe: two LysR-type transcription factors mediate oxidative stress protection

An intriguing question arises: Why does *Agrobacterium* possess two thiol-based redox sensors of the LTTR family to combat oxidative stress? We propose that this dual regulatory system functions as a “fail-safe” mechanism, ensuring robust and efficient management of oxidative stress, thereby supporting proliferation during pathogenesis (Fig. 11).

We hypothesize that OxyR acts as a “fast” sensor, rapidly responding to subtle shifts in the intracellular redox state. This is supported by a previous study demonstrating *katG* induction at low H_2_O_2_ concentrations (250 μM) [24]. OxyR activity is tightly controlled by oxidation-dependent autoregulation and by LsrB. In contrast, *lsrB* is constitutively expressed, suggesting a continuous need for rapid adaptation to changing environmental conditions (Supplementary Fig. S3). The large regulon of LsrB [36] suggests a broader role beyond regulating antioxidant genes, potentially functioning as a master stress response regulator. Supporting this notion, LsrB influences a broad range of cellular processes, including β-lactam resistance [37]. Notably, bactericidal antibiotics can induce ROS formation, such as hydrogen peroxide and hydroxyl radicals, through increased repair demands and subsequent NADH depletion [102]. Hydroxyl radical formation is tightly linked to enhanced Fenton chemistry, which could explain why LsrB regulates iron-sequestration genes.

The second reason for having two redox-responsive LTTRs may be that if one regulator becomes over-oxidized, the other can at least partially compensate, ensuring continued oxidative protection. This functional redundancy in antioxidant regulation likely provides a substantial adaptive advantage in complex and frequently changing environments. Third, the distinct genomic locations of *oxyR* and *lsrB* increase the likelihood that at least one will be successfully transcribed, even when ROS-induced DNA damage is extensive. In summary, our findings suggest that the interplay between LsrB and OxyR equips *A. tumefaciens* with a robust and adaptable oxidative stress response system.

## Supplementary Material

gkaf267_Supplemental_Files

## Data Availability

The RNA-seq data are available at the at Gene Expression Omnibus database at https://www.ncbi.nlm.nih.gov/geo/ under the accession number GSE283062. The mass spectrometry data are deposited at the ProteomeXchange Consortium via the PRIDE partner repository (https://www.ebi.ac.uk/pride/) under the dataset identifier PXD055627. The cryo-EM structure data are deposited at the Protein Data Bank (PDB, https://www.rcsb.org/) under the accession ID 9HH1.

## Notes

present address: division of molecular and cellular biology, eunice kennedy shriver national institute of child health and human development, bethesda, md 20892, usa
