# Supplementary material for: Two redox-responsive LysR-type transcription factors control the oxidative stress response of Agrobacterium tumefaciens
Source: Nucleic Acids Res. 2025 Apr 7;53(6):gkaf267. doi: 10.1093/nar/gkaf267 (PMC11975290; doi:10.1093/nar/gkaf267)
Supplement: gkaf267_Supplemental_Files [file gkaf267_supplemental_files.zip › Supplement_Schmidt_final.pdf]

Two redox-responsive LysR-type transcription factors control the oxidative stress response of *Agrobacterium tumefaciens*

-SUPPLEMENT-

Janka J. Schmidt[1,8], Vivian B. Brandenburg[2], Hannah Elders[3], Saba Shahzad[4], Sina Schäkermann[5,6], Ronja Fiedler[1], Lisa R. Knoke[7], Yvonne Pfänder[1], Pascal Dietze[5], Hannah Bille[1], Bela Gärtner[1], Lennart J. Albin[1], Lars I. Leichert[7], Julia E. Bandow[5,6], Eckhard Hofmann[3], & Franz Narberhaus[1]*

[1]Microbial Biology, Ruhr University Bochum, 44801 Bochum, Germany

[2]Bioinformatics Group, Ruhr University Bochum, 44801 Bochum, Germany

[3]Protein Crystallography, Ruhr University Bochum, 44801 Bochum, Germany

[4]Ernst Ruska-Centre for Microscopy and Spectroscopy with Electrons (ER-C-3): Structural Biology, Institute of Biological Information Processing (IBI-6): Structural Cell Biology, Forschungszentrum Jülich, 52428 , Germany.

[5]Applied Microbiology, Ruhr University Bochum, 44801 Bochum, Germany

[6]Center for System-based Antibiotic Research, Ruhr University Bochum, 44801 Bochum, Germany

[7]Microbial Biochemistry, Ruhr University Bochum, 44801 Bochum, Germany

[8]Present address: Division of Molecular and Cellular Biology, Eunice Kennedy Shriver National Institute of Child Health and Human Development, Bethesda, MD 20892, USA.

Running title: Redox regulators in *Agrobacterium tumefaciens*

Key words: Oxidative stress, LTTR, OxyR, plant-microbe interaction, cryo-EM, transcriptional regulation

*For correspondence. E-mail franz.narberhaus@rub.de; Tel. (+49) (234) 322 3100; Fax (+49) (234) 321 4620.

SUPPLEMENT

Table S1: Strains

Strain	Description	Source
E. coli JM83	Cloning host	[1]
E. coli DH5α	Cloning host	[2]
E. coli BL21 (DE3) + pPB176	*E. coli* strain with pPB176 coding for *lsrB*^{His} (*atu2186*) (pET derivate) under the control of a *lac* promotor. Km^{R}	[3]
E. coli BL21 (DE3) + pRNA73	*E. coli* strain with pRNA73 coding for *lsrB*^{Strep} (*atu2186*) (pASK-IBA3) under the control of a *tet* promotor. Amp^{R}	[3]
E. coli BL21 (DE3) + pRF10	*E. coli* strain with pRF10 coding for ^{His}*argP (eck2912)* (pCA24M derivate/ ASKA collection) under the control of a *lac* promotor. Cm^{R}	[3]
A. tumefaciens C58	WT strain	C. Baron, Montréal, Canada
A. tumefaciens C58 + pSRK	WT strain with empty vector. Km^{R}	[4]
A. tumefaciens C58 Δ*lsrB*	Knock-out of *lsrB* (*atu2186*) in *A. tumefaciens* C58. Gm^{R}	[5]
A. tumefaciens C58 Δ*lsrB* + pSRK	Knock-out of *lsrB* (*atu2186*) in *A. tumefaciens* C58 with empty vector (pSRK). Km^{R}	[4]
A. tumefaciens C58 Δ*lsrB* + p*lsrB*	Δ*lsrB* mutant with plasmid encoded *lsrB*^{StrepII}. Km^{R}, Gm^{R}	[4]
A. tumefaciens C58 Δ*oxyR*	Markerless deletion of *atu4641* with pJS51	This study
A. tumefaciens C58 Δ*lsrB/* Δ*oxyR*	Markerless deletion of *atu4641* with pJS51 in *A. tumefaciens ΔlsrB.* Gm^{R}	This study
A. tumefaciens C58 Δ*lsrB* + p*lsrB* (C144S)	Δ*lsrB* mutant with plasmid encoded *lsrB*^{StrepII}. Km^{R}, Gm^{R} (pJS06)	This study
A. tumefaciens C58 Δ*lsrB* + p*lsrB* (C236S)	Δ*lsrB* mutant with plasmid encoded *lsrB*^{StrepII}. Km^{R}, Gm^{R} (pJS11)	This study
A. tumefaciens C58 Δ*lsrB* + p*lsrB* (C244S)	Δ*lsrB* mutant with plasmid encoded *lsrB*^{StrepII}. Km^{R}, Gm^{R} (pJS09)	This study
A. tumefaciens C58 Δ*lsrB* + p*lsrB* (C273S)	Δ*lsrB* mutant with plasmid encoded *lsrB*^{StrepII}. Km^{R}, Gm^{R} (pJS12)	This study
A. tumefaciens C58 Δ*lsrB* + p*lsrB* (C144S/C273S)	Δ*lsrB* mutant with plasmid encoded *lsrB*^{StrepII}. Km^{R}, Gm^{R} (pJS15)	This study
A. tumefaciens C58 Δ*lsrB* + p*lsrB* (C236S/C244S)	Δ*lsrB* mutant with plasmid encoded *lsrB*^{StrepII}. Km^{R}, Gm^{R} (pJS16)	This study
A. tumefaciens C58 Δ*lsrB* + p*lsrB* (C144S/ C244S/C273S)	Δ*lsrB* mutant with plasmid encoded *lsrB*^{StrepII}. Km^{R}, Gm^{R} (pJS38)	This study
A. tumefaciens C58 Δ*lsrB* + p*lsrB* (C236S/ C244S/C273S)	Δ*lsrB* mutant with plasmid encoded *lsrB*^{StrepII}. Km^{R}, Gm^{R} (pJS39)	This study
A. tumefaciens C58 Δ*lsrB* + p*lsrB* (C144S/ C236S/C244S)	Δ*lsrB* mutant with plasmid encoded *lsrB*^{StrepII}. Km^{R}, Gm^{R} (pJS40)	This study
A. tumefaciens C58 Δ*lsrB* + p*lsrB* (C144S/ C244S/C273S)	Δ*lsrB* mutant with plasmid encoded *lsrB*^{StrepII}. Km^{R}, Gm^{R} (pJS41)	This study
A. tumefaciens C58 Δ*lsrB* + p*lsrB* (C144S/ C236S/C244S/C273S)	Δ*lsrB* mutant with plasmid encoded *lsrB*^{StrepII}. Km^{R}, Gm^{R} (pJS24)	This study

A. tumefaciens C58 + pBISN1	WT with pBISN1. Km^R	This study
A. tumefaciens C58 Δ*lsrB* + pBISN1	Δ*lsrB* with pBISN1. Km^R	This study
A. tumefaciens C58 Δ*oxyR* + pBISN1	Δ*oxyR* with pBISN1. Km^R	This study
A. tumefaciens C58 Δ*lsrB/* Δ*oxyR* + pBISN1	Δ*lsrB/* Δ*oxyR* with pBISN1. Km^R	This study
A. tumefaciens C58 + pLK23	WT strain with pTrc200 with *grx1-roGFP2*. $Strep^R$, $Spec^R$ (pLK23)	This study
A. tumefaciens C58 Δ*lsrB* + pLK23	Δ*lsrB* strain with pTrc200 with *grx1-roGFP2*. $Strep^R$, $Spec^R$ (pLK23)	This study

Table S2: Plasmids

Plasmid	Description	Source
pK19*mobsacB*	Suicide vector (Cloning vector). Km^R	[6]
pSRK	Complementation vector. Km^R	[7]
pTrc200	Expression vector. $Strep^R$, $Spec^R$, trc promoter	[8]
pBISN1	Expression vectore with *gusA*-intronvector. Km^R	[9]
pET28a	Expression vector. Km^R. T7 promoter	Addgene
pRF10	pRF10 coding for ^{His}*argP* (pCA24M derivate/ ASKA collection) under the control of a *lac* promotor. Cm^R	[3]
p*lsrB*	Complementation plasmid with *lsrB (atu2186)*. Constructed from pSRK.	[4]
pB176	Expression vector with *lsrB*^{His} under the control of a T7 promotor. Km^R	[3]
pRNA72	*Expression vector with lsrB*^{Strep} under control of a tet promotor. Amp^R	This study
pJSS89 (p*katG*)	Complementation plasmid with *katG^{His} (atu4642)*. Constructed from pTRC200.	This study
pLK23	Expression plasmid with *grx1-roGFP2*. Constructed from pTrc200	This study
pJS51	For markerless deletion of *oxyR* (*atu4641)*, constructed from pK19*mobsacB*	This study
pJS06	Complementation plasmid with *lsrB* (*atu2186*) with C144S via site-directed mutagenesis. Constructed from p*lsrB.*	This study
pJS09	Complementation plasmid with *lsrB* (*atu2186*) with C244S via site-directed mutagenesis. Constructed from p*lsrB.*	This study
pJS11	Complementation plasmid with *lsrB* (*atu2186*) with C236S via site-directed mutagenesis. Constructed from p*lsrB.*	This study
pJS12	Complementation plasmid with *lsrB* (*atu2186*) with C273S via site-directed mutagenesis. Constructed from p*lsrB.*	This study
pJS15	Complementation plasmid with *lsrB* (*atu2186*) with C144S + C273S via site-directed mutagenesis. Constructed from p*lsrB.*	This study
pJS16	Complementation plasmid with *lsrB* (*atu2186*) with C236S + C244S via site-directed mutagenesis. Constructed from p*lsrB.*	This study
pJS24	Complementation plasmid with *lsrB* (*atu2186*) with C144S, C236S, C244S and C273s synthesized via Twist Bioscience. Constructed from p*lsrB.*	This study
pJS38	Complementation plasmid with *lsrB* (*atu2186*) with C144S, C244S and C273S. Constructed from p*lsrB.*	This study
pJS39	Complementation plasmid with *lsrB* (*atu2186*) with C236S, C244S and C273S. Constructed from p*lsrB.*	This study
pJS40	Complementation plasmid with *lsrB* (*atu2186*) with C144S, C236S and C244S. Constructed from p*lsrB.*	This study
pJS41	Complementation plasmid with *lsrB* with C144S, C44S and C244S. Constructed from p*lsrB.*	This study

Table S3: Oligonucleotides

Primer name	Sequence	Designated use
oxyR fragment 1 fw	GCGGATCCCATGGTGGCCGACAAGTTCG	Deletion *oxyR*
oxyR fragment 1 rv	GCAAGCTTCGCAGATGGCGGTCGATATG	
oxyR fragment 2 fw	GCAAGCTTCAGATCCTGCAATTCGTCCAC	
oxyR fragment 2 rv	GCGAATTCTGCTGCGATGCCGATGATCAT	
grx1 fw	AAACCATGGCTCAAGAGTTTGTGAAC	roGFP probe
roGFP-2 rev	AAATCTAGATTACTTGTACAGCTCGTCC	
C144S fw	ATGCCGATAGCGCCATCCGCCTGCGCC	Site-directed mutagenesis
C144S rv	GGATGGCGCTATCGGCATGGCGCATGTTCA	
C236S fw	GCGCGAGCCTGCTGGGGATTGGTATTGC	
C236S rv	AGCAGGCTCGCGCGCTTGATCGAGGTCT	
C244S fw	GTATTGCCAGTTTGCCCGACTATATCGTCGG	
C244S rv	TCGGGCAAACTGGCAATACCAATCCCCAG	
C273S fw	CGATACCTATTTCAGCTATCCCGACGAGATG	
C273S rv	CGGGATAGCTGAAATAGGTATCGAAGGACGG	
katG qRT fw	CGAACCAACCAAGTGGGACAACG	qRT-PCR
katG qRT rv	CAGGCTTGTCTTCTTCGGCGATG	
dps qRT fw	GATCGAGCGTTATGGCGAAGTCG	
dps qRT rv	CACAGTGACTTGTCGAGATCGCG	
ohr qRT fw	CGGCGACAAGTTCTTCGGCTTTC	
ohr qRT rv	GACAATGGAAAGCCTGTTGAGGC	
lsrB qRT fw	GACCTTATCCAGCGCAAGCTGTTC	
lsrB qRT rv	CCAAAGGAGATGATGCGGTGATTGTC	
oxyR qRT fw	CGTCACGCTTATTCCGCAGATGG	
oxyR qRT rv	GATTGCTCTTGCGCCAGACGATG	
gyrB qRT fw	AGGTCACCTCCTATGCCGAT	
gyrB qRT rv	CGGGAACCTTGACCGAAAGA	
rpoA qRT fw	ACGGGTGTTTTCCACCTTGT	
rpoA qRT rv	CAACAACGGCAAGGGTTACG	
katG EMSA fw	CTCAGCATATCGCAGCCTTTAGA	EMSA
katG EMSA rv	GCCGGTTTTGAAGTTGCGTCC	
ampC EMSA fw	GCGGCTAAACGTCTTTTCGC	
ampC EMSA rv	CATCATCAGCGGCAGAAGC	
lysP EMSA fw*	CGCTTTCTGGACTATTGCGATC	
lysP EMSA rv*	CGCTTCTGTGGTTTTAGTTTCG	

*Primer sequences were obtained from [10]

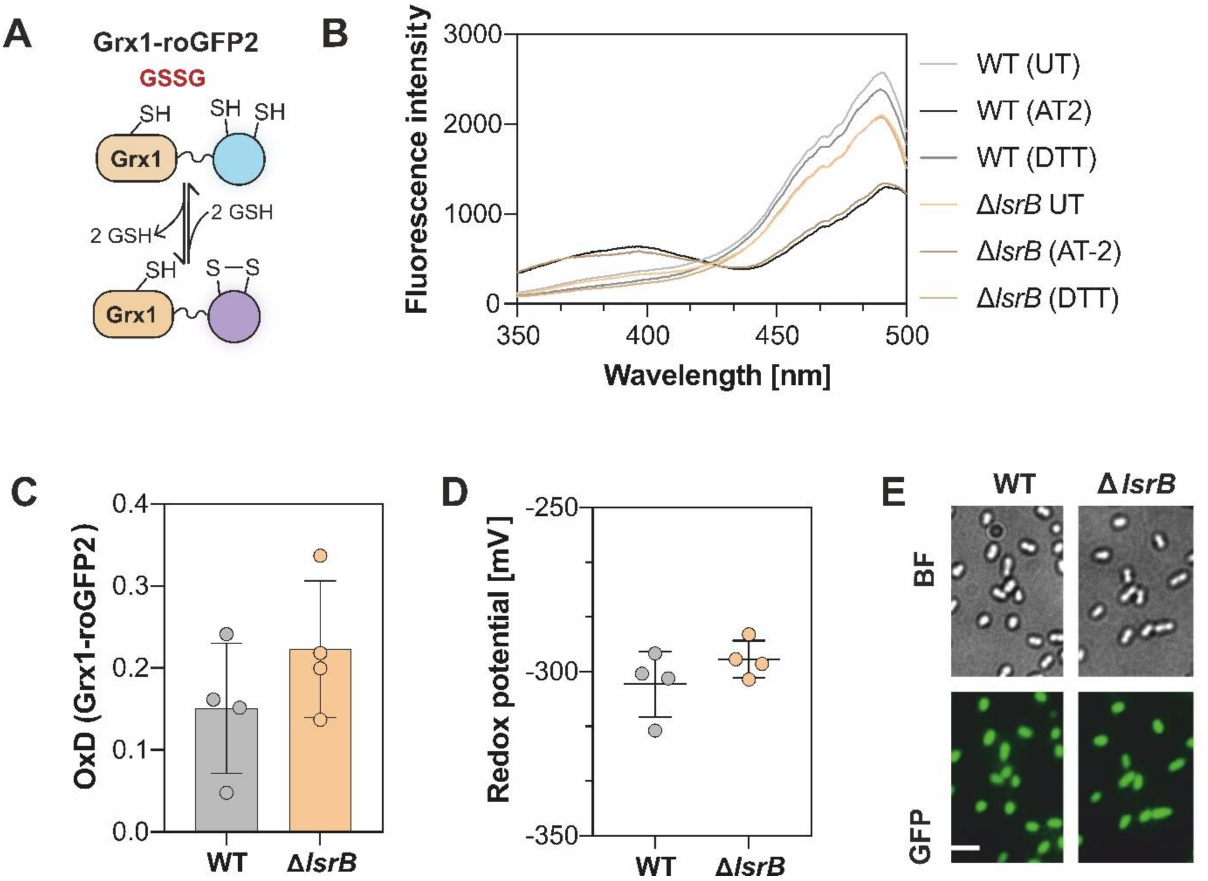

Figure S1. Effect of LsrB on the glutathione homeostasis and redox potential in *Agrobacterium tumefaciens*. A The Grx1-roGFP2 probe specifically measures the cellular glutathione homeostasis. **B** Excitation spectra of reduced (DTT), oxidized (AT-2) and untreated cells. The spectra were recorded at 20 °C in PBS buffer with emission at 510 nm. **C** Oxidation state (OxD) of Grx1-roGFP2 in *A. tumefaciens* WT and *A. tumefaciens* Δ*lsrB*. The OxD was determined based on samples reduced with DTT and oxidized with Aldrithiol-2 (AT-2) from the ratio of the fluorescence intensities measured at 405 and 488 nm excitation. Each value represents one individual experiment and error bars are the standard deviation in n = 4 individual replicates. **D** Redox potential exerted on Grx1-roGFP2 in *A. tumefaciens* WT and *A. tumefaciens* Δ*lsrB*. The steady state redox potential was calculated using the Nernst equation and the OxD values from (C). **E** Expression and localization of Grx1-roGFP2 is confirmed by fluorescence microscopy.

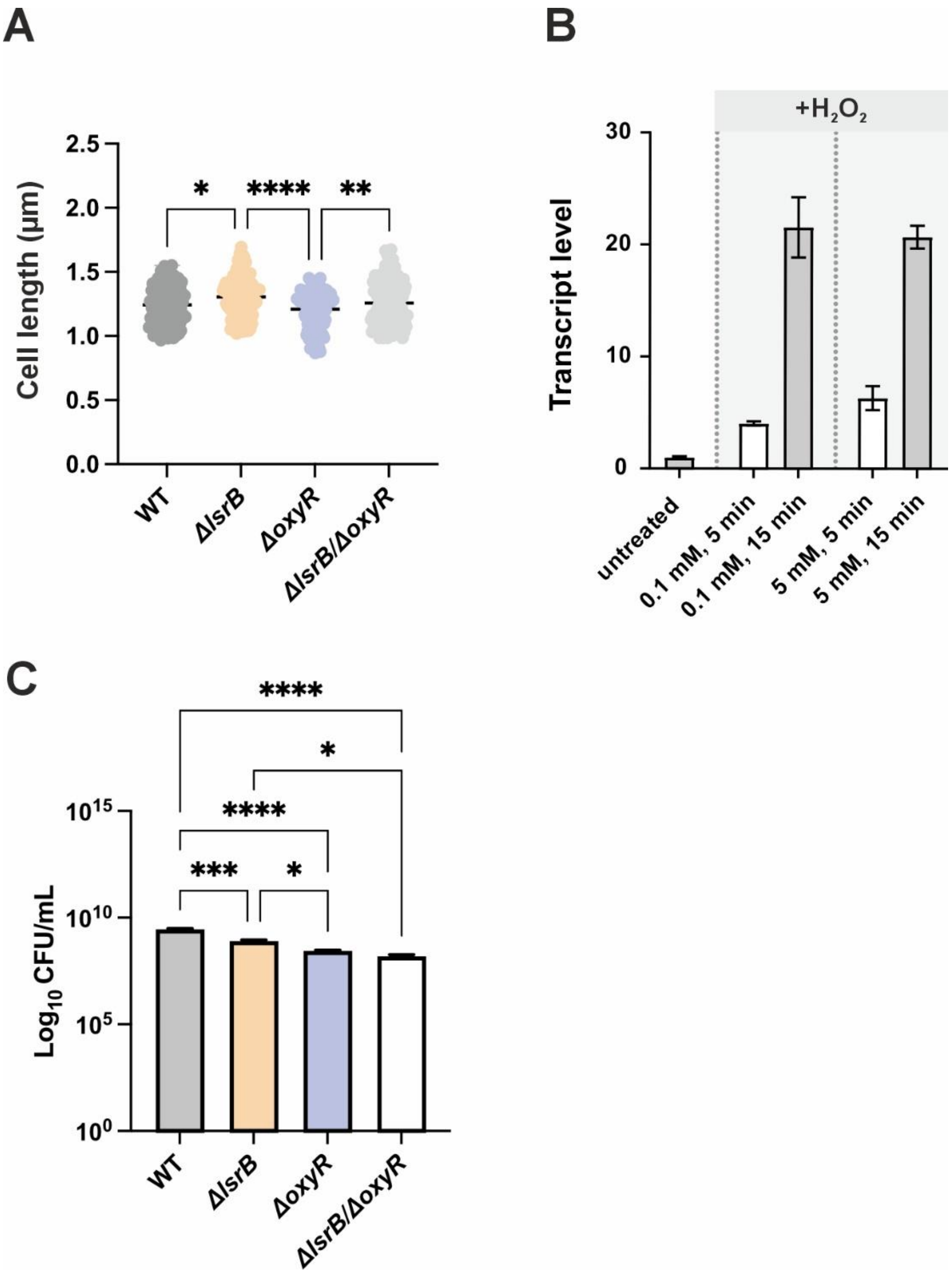

Figure S2. Phenotypic effects of *lsrB* and *oxyR* deletion. A Cell length measurement of *A. tumefaciens* WT, *ΔlsrB, ΔoxyR* and *ΔlsrBΔoxyR.* Cells were cultivated in LB medium until mid-exponential phase. Error bars represent ± mean standard deviation of four replicates (n=100). Significance was tested by one-way ANOVA**.** *p<0.05, **p < 0.01, ***p < 0.001, ****p < 0.0001. **B** *katG* expression levels after H_2O_2 stress. Transcript levels of *katG* in *A. tumefaciens* WT, grown in LB, to mid exponential phase +/- H_2O_2 exposure (0.1 or 5 mM for 5 or 15 min). Transcript levels were normalized to *gyrB* and *rpoA* levels. Error bars represent ± mean standard deviation of three technical replicates each. **C** H_2O_2 survival. CFU were analyzed after 15 min, 5 mM H_2O_2 exposure. Cells exposed to H_2O_2 were diluted 10^{-6}, plated on LB plates and incubated at 30°C for 48 h. Error bars represent ± standard deviation of two independent experiments.

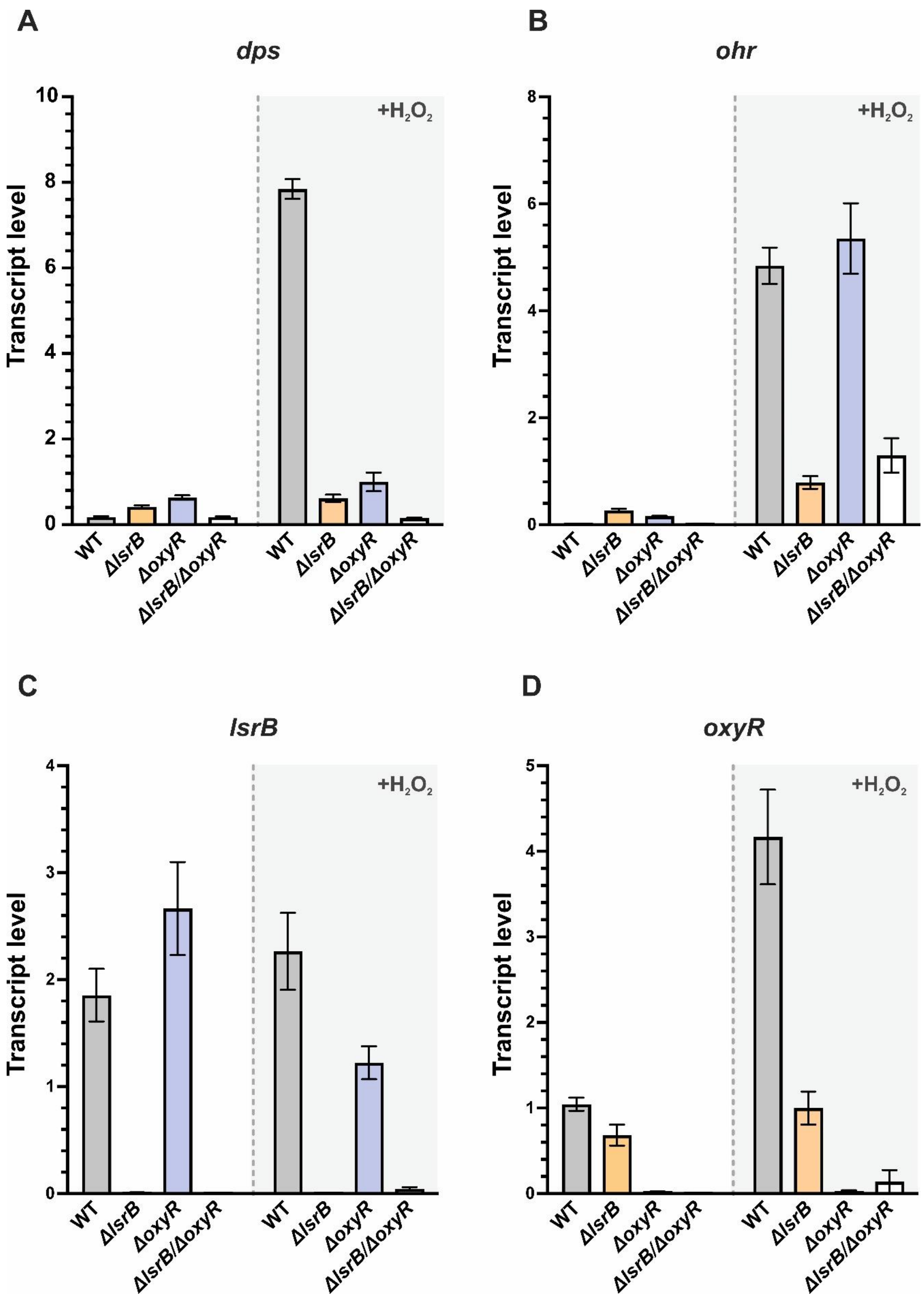

Figure S3. Transcript levels of selected genes before and after H_2O_2 stress. *A. tumefaciens* strains were cultivated in LB-medium to mid exponential phase and harvested prior or after H_2O_2 exposure for 15 min. Transcript levels of **A** *dps*, **B** *ohr*, **C** *lsrB* and **D** *oxyR* were examined via qRT-PCR. Transcript levels were normalized to *gyrB*-levels (reference gene). Error bars represent ± standard error of the mean (SEM) of three independent replicates.

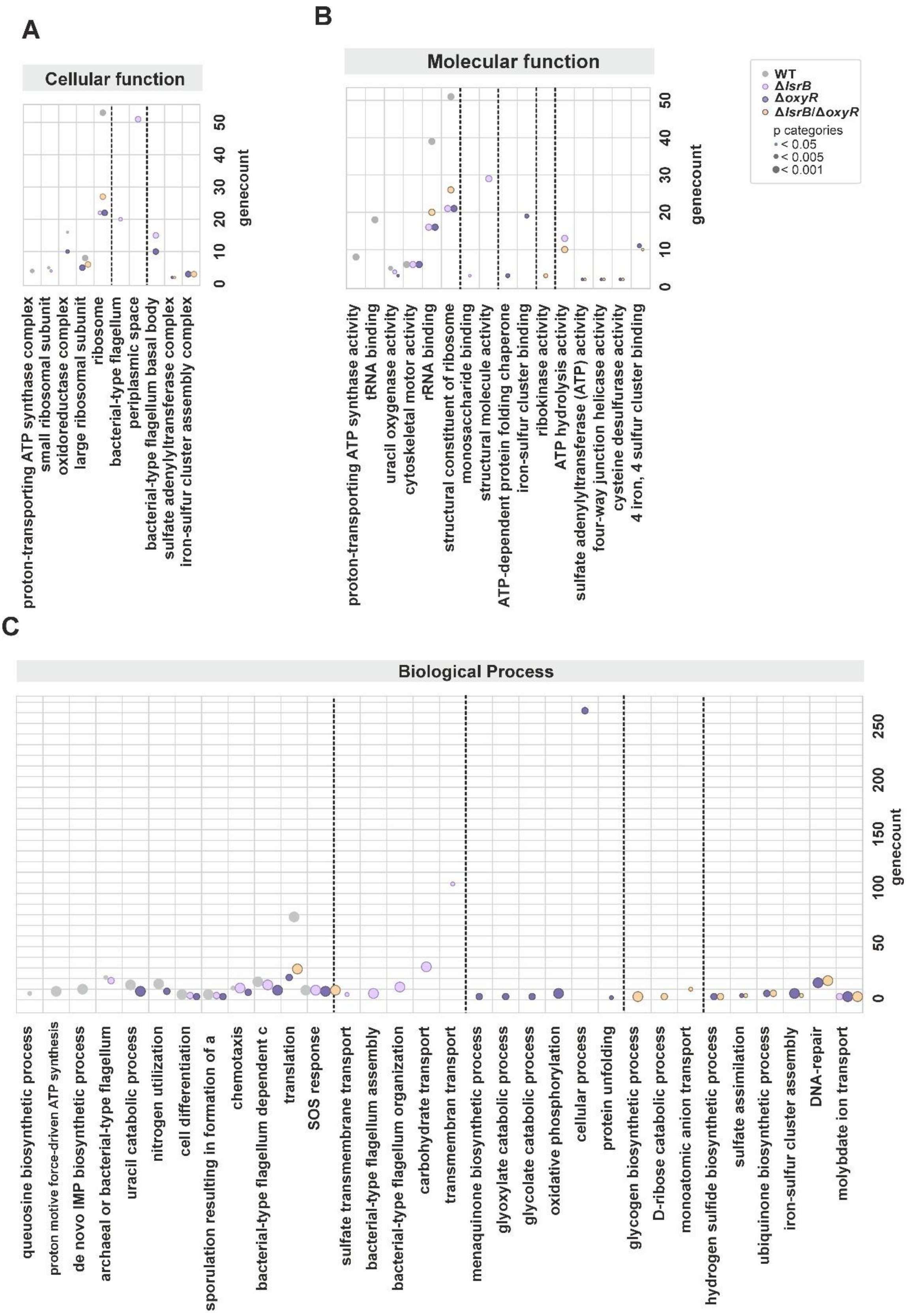

Figure S4. Gene ontology enrichment analysis. Gene ontology (GO) term enrichment of differentially regulated genes in A. *tumefaciens* WT and the *ΔlsrB*, *ΔoxyR* and *ΔlsrB/ΔoxyR* mutants during hydrogen peroxide stress ($log_2FC > 1$, p-value < 0.01). Horizontal coordinates represent GO terms, vertical coordinates the total number of differentially regulated genes in response to hydrogen peroxide. The number of enriched genes according to cellular function (**A**), molecular function (**B**) and biological processes (**C**). The x-axis denotes the total number of significantly enriched genes within each GO term. The size and color of the data points represent the genes and p-values associated with the respective GO term.

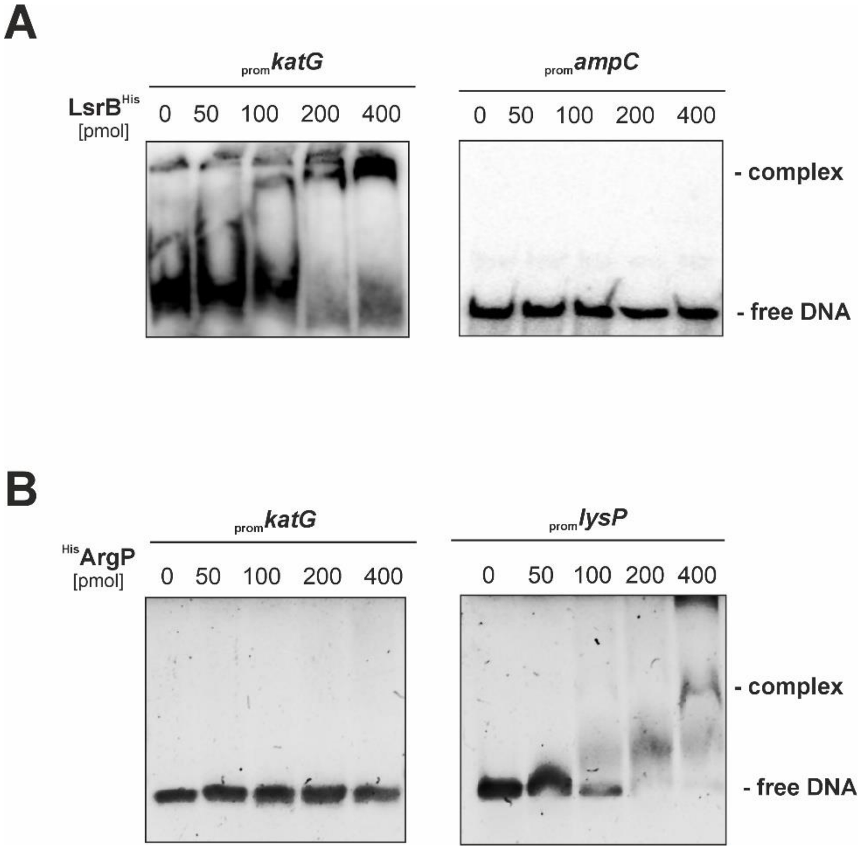

Figure S5: LsrB specifically binds to the *katG* promoter region. EMSAs showing DNA-protein interactions. **A** A 125-bp fragment upstream of the *katG* coding sequence was labeled with ^{32}P and incubated with increasing concentrations of recombinantly purified $LsrB^{His}$ (left), whilst a 155-bp fragment upstream of *ampC* served as a non-binding control (right), as published previously [3]. Herring sperm DNA was used as a competitor. The protein-DNA complex and free promoter DNA are indicated. **B** Non-radioactive EMSA as negative control with the LTTR $^{His}ArgP$. A 276-bp fragment upstream of *lysP* [10] was used as a positive control to confirm ArgP functionality. All EMSAs were performed in three independent replicates, with one representative shown.

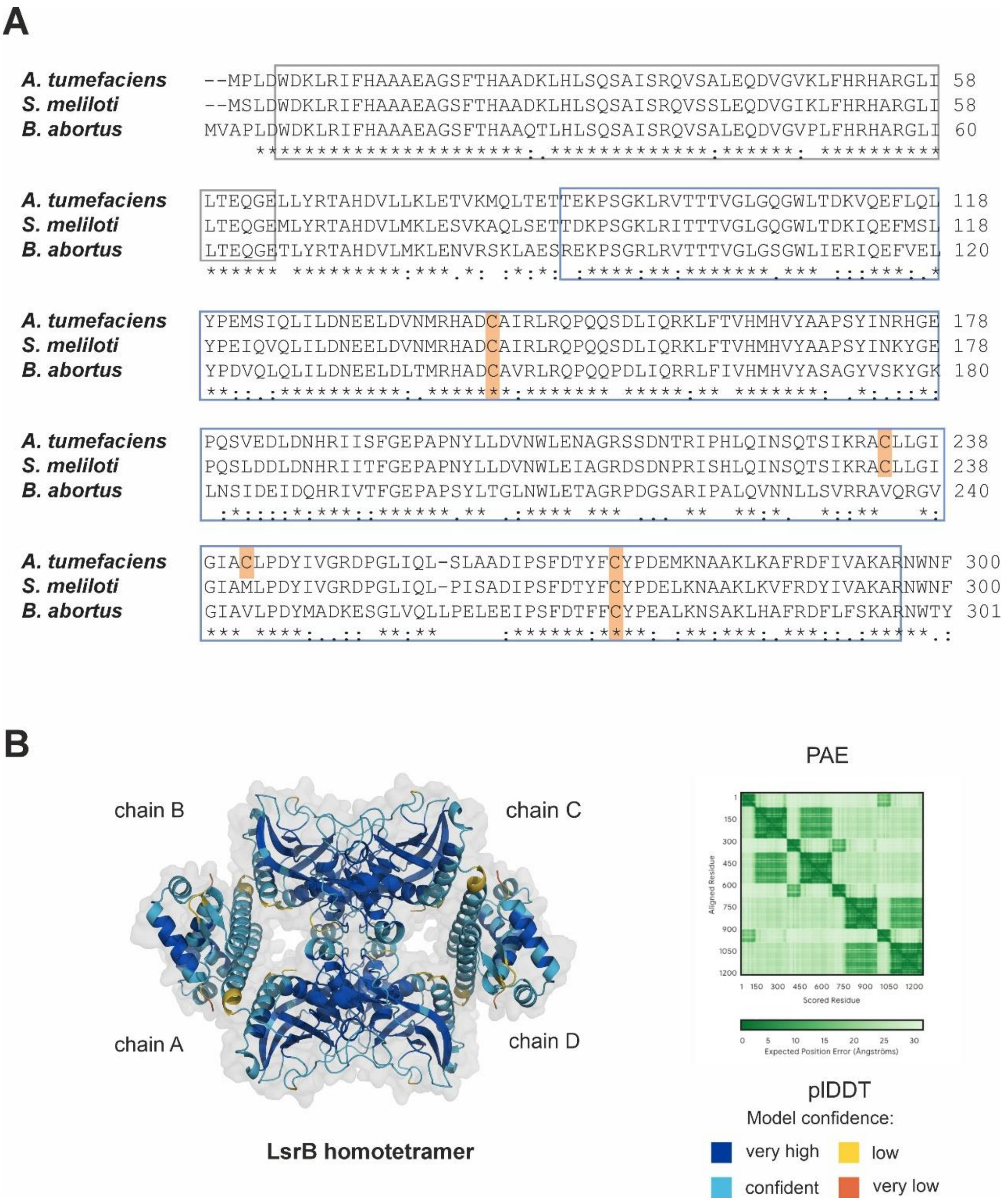

Figure S6: LsrB protein sequence and structure. A Amino acid sequence alignment of LsrB proteins. LsrB from *A. tumefaciens* (A9CI74), *S. meliloti* (Q92PZ7*) or B. abortus* (Q2YRP). Sequence alignment was calculated by Clustal Omega[11]. The N-terminal DNA-binding domain is in grey borders, C-terminal co-effector binding domain is bordered in blue. Cysteine residues are highlighted in orange. Identical amino acid residues (*), residues with high similarity (:) and low similarity. **B** 3D structure model of LsrB (A9CI74). The structure model was generated by AlphaFold 3 [12,13]**.** The plDDT (per-residue model confidence score) is indicated by color. PAE = Expected Position Error.

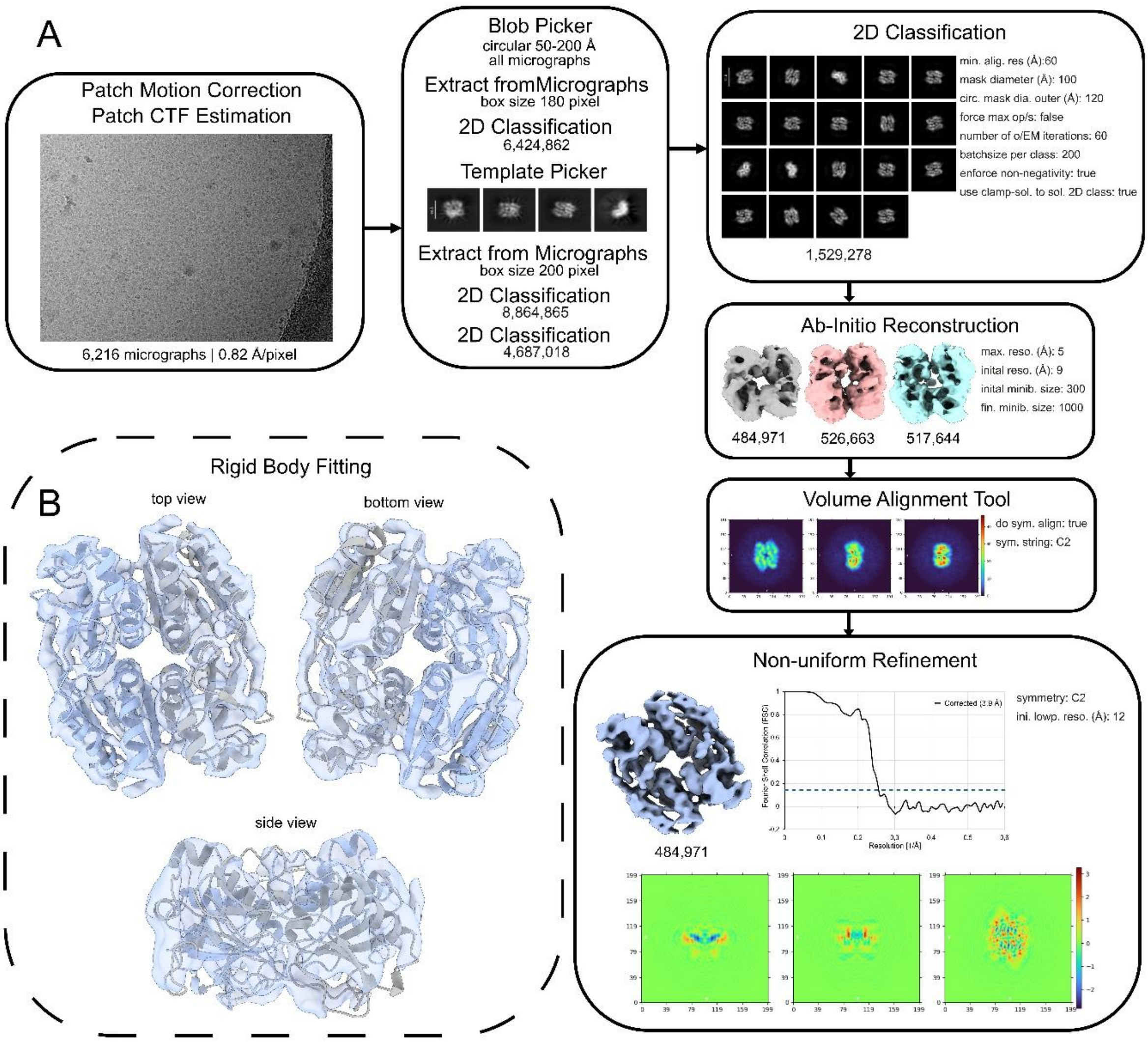

Figure S7. Image processing workflow for Strep-tagged LsrB SBD dimer. A Workflow of jobs used for volume reconstruction and refinement in CryoSPARC [14]. All jobs are indicated with their respective names. Selected and used particles for each job are indicated in each box. All jobs were run with default parameters unless given in the boxes. The graph shows the Fourier Shell Correlation (FSC) for the final map of the Non-uniform Refinement. The corrected FSC values were used, and the global resolution was estimated by the gold-standard FSC.**B** AlphaFold 3 model of the LsrB^{Strep} SBD dimer fitted into the reconstructed volume with rigid body fitting using UCSF Chimera [15].

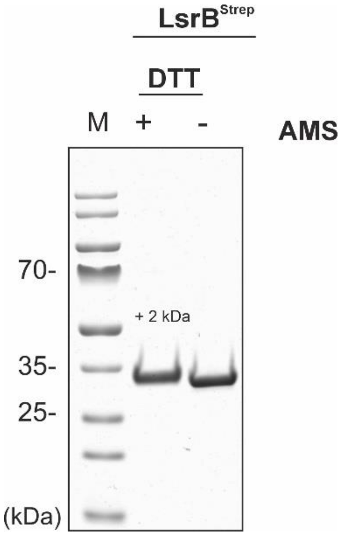

Figure S8: AMS in-vitro alkylation of free thiols. Recombinantly purified LsrB was reduced using a 10-fold molar access of DTT, prior to alkylation with AMS (4-acetamido-4'-maleimidylstilbene-2,2'-disulfonic acid) or water (control). The observed band shift in the AMS-treated sample indicates successful alkylation of accessible thiols, as AMS addition increases the protein's molecular weight by ca. 0.5 kDa. Experiments were performed in three independent replicates.

References

1. Vieira J, Messing J. The pUC plasmids, an M13mp7-derived system for insertion mutagenesis and sequencing with synthetic universal primers. *Gene*. 1982;19(3):259-268. doi:10.1016/0378-1119(82)90015-4
2. Woodcock DM, Crowther PJ, Doherty J, et al. Quantitative evaluation of *Escherichia coli* host strains for tolerance to cytosine methylation in plasmid and phage recombinants. *Nucleic Acids Res*. 1989;17(9):3469. doi:10.1093/NAR/17.9.3469
3. Schmidt JJ, Remme DCLE, Eisfeld J, Brandenburg VB, Bille H, Narberhaus F. The LysR-type transcription factor LsrB regulates beta-lactam resistance in *Agrobacterium tumefaciens*. *Mol Microbiol*. 2024;121(1):26-39. doi:10.1111/MMI.15191
4. Eisfeld J, Kraus A, Ronge C, Jagst M, Brandenburg VB, Narberhaus F. A LysR-type transcriptional regulator controls the expression of numerous small RNAs in Agrobacterium tumefaciens. *Mol Microbiol*. 2021;116(1):126-139. doi:10.1111/MMI.14695
5. Kraus A, Weskamp M, Zierles J, et al. Arginine-rich small proteins with a domain of unknown function, DUF1127, play a role in phosphate and carbon metabolism of *Agrobacterium tumefaciens*. *J Bacteriol*. 2020;202(22). doi:10.1128/JB.00309-20
6. Schäfer A, Tauch A, Jäger W, Kalinowski J, Thierbach G, Pühler A. Small mobilizable multi-purpose cloning vectors derived from the *Escherichia coli* plasmids pK18 and pK19: selection of defined deletions in the chromosome of Corynebacterium glutamicum. *Gene*. 1994;145(1):69-73. doi:10.1016/0378-1119(94)90324-7
7. Khan SR, Gaines J, Roop RM, Farrand SK. Broad-host-range expression vectors with tightly regulated promoters and their use to examine the influence of TraR and TraM expression on Ti Plasmid quorum sensing. *Appl Environ Microbiol*. 2008;74(16):5053-5062. doi:10.1128/AEM.01098-08
8. Schmidt-Eisenlohr H, Domke N, Angerer C, Wanner G, Zambryski PC, Baron C. Vir proteins stabilize VirB5 and mediate its association with the T pilus of *Agrobacterium tumefaciens*. *J Bacteriol*. 1999;181(24):7485-7492. doi:10.1128/JB.181.24.7485-7492.1999
9. Narasimhulu SB, Deng XB, Sarria R, Gelvin SB. Early transcription of *Agrobacterium* T-DNA genes in tobacco and maize. *Plant Cell*. 1996;8(5):873-886. doi:10.1105/tpc.8.5.873
10. Ruiz J, Haneburger I, Jung K. Identification of ArgP and Lrp as transcriptional regulators of *lysP*, the gene encoding the specific Lysine permease of *Escherichia coli*. *J Bacteriol*. 2011;193(10):2536-2548. doi:10.1128/JB.00815-10
11. Sievers F, Wilm A, Dineen D, et al. Fast, scalable generation of high-quality protein multiple sequence alignments using Clustal Omega. *Mol Syst Biol*. 2011;7. doi:10.1038/MSB.2011.75
12. Abramson J, Adler J, Dunger J, et al. Accurate structure prediction of biomolecular interactions with AlphaFold 3. *Nature 2024*. Published online May 8, 2024:1-3. doi:10.1038/s41586-024-07487-w
13. Abramson J, Adler J, Dunger J, et al. Addendum: Accurate structure prediction of biomolecular interactions with AlphaFold 3. *Nature*. Published online November 27, 2024. doi:10.1038/s41586-024-08416-7
14. Punjani A, Rubinstein JL, Fleet DJ, Brubaker MA. cryoSPARC: algorithms for rapid unsupervised cryo-EM structure determination. *Nature Methods 2017 14:3*. 2017;14(3):290-296. doi:10.1038/nmeth.4169
15. Meng EC, Goddard TD, Pettersen EF, et al. UCSF ChimeraX: Tools for structure building and analysis. *Protein Sci*. 2023;32(11). doi:10.1002/PRO.4792
